# Bird conservation status and cultural values in Indigenous Mexican communities: towards a bioculturally informed conservation policy

**DOI:** 10.1186/s13002-022-00567-z

**Published:** 2022-12-02

**Authors:** Graciela Alcántara-Salinas, Eugene S. Hunn, María Elena Ibáñez-Bravo, Elda Miriam Aldasoro-Maya, Noé Flores-Hernández, Juan Antonio Pérez-Sato, Natalia Real-Luna, Rafael Arturo Muñoz-Márquez Trujillo, Diana Lope-Alzina, Jaime Ernesto Rivera-Hernández

**Affiliations:** 1 Colegio de Postgraduados Campus Córdoba (COLPOS), Carretera Federal-Veracruz km. 348, Congregación Manuel León, Amatlán de los Reyes, C.P. 94953 Córdoba, Veracruz Mexico; 2 University of Washington, 314 Denny Hall, Box 353100, Seattle, WA USA; 3 Escuela Nacional de Antropología e Historia (ENAH), Periférico Sur y Calle Zapote s/n, Isidro Fabela, C. P. 14030, Tlalpan, Mexico City, Mexico; 4 El Colegio de la Frontera Sur (ECOSUR), Periférico Sur s/n, María Auxiliadora, C.P. 29290 San Cristóbal de las Casas, Chiapas Mexico; 5 Escuela Nacional de Trabajo Social, Universidad Nacional Autónoma de México (UNAM), Circuito exterior s/n, Ciudad Universitaria, Alcaldía de Coyoacán, C.P. 04510 Coyoacán, Mexico City, Mexico; 6 Alliance Bioversity-CIAT, Via di S. Domenico 1, 00153 Roma, Italia; 7 Centro de Estudios Geográficos, Biológicos y Comunitarios, S.C. (GEOBICOM), Santa María No. 13 U.H. San Román, C.P. 94542 Córdoba, Veracruz Mexico

**Keywords:** Ethnoornithology, Mexican Indigenous communities, Nomenclatural recognition, Cultural values of birds, Biocultural conservation policies

## Abstract

**Background:**

We summarize comparative ethnoornithological data for ten Mexican Indigenous communities, an initial step towards a comprehensive archive of the avian diversity conserved within Mexico’s Indigenous territories. We do so by counting highlighted species listed for bird conservation status on widely recognized “red lists” and their cultural value to build biocultural policies in Mexico for their conservation.

**Methods:**

Indigenous bird names for each study site were determined to allow calculation of the “Scientific Species Recognition Ratio” (SSRR) for high cultural value birds obtained across communities. This demonstrated patterns of cultural prominence. A matrix of 1275 bird versus seven biocultural values was analysed using a correspondence analysis (InfoStat/L-v2020) to illustrate patterns of concordance between bird conservation status and cultural values.

**Results:**

This paper contributes to quantitative and qualitative data on the role of ethnoornithology and ethnobiology in biocultural conservation. The areas studied provide refugia for almost 70% of the Mexican avifauna within a fraction of 1% of the national territory, that is 769 bird species recorded for all communities. The global correspondence of regions of biological and linguistic megadiversity is well established, while linguistic diversity is widely accepted as a good proxy for general cultural diversity. Our correspondence analysis explained 81.55% of the variation, indicating a strong relation between cultural importance and bird conservation status. We propose three main categories to establish a bioculturally informed public policy in Mexico for the conservation of what we described as high, medium, and bioculturally prominent bird species all include cultural value in any material or symbolic aspect. High are those species appearing on any threatened list, but also considered in any endemic status, while medium include threatened listed species. The last category included species not necessarily listed on any threat list, but with a wide range of social and cultural uses. We suggest that the concept might be extended to other species of biocultural importance.

**Conclusions:**

We argue that bird conservation policies should be biocultural, that is they should recognize birds of cultural value on a par with bird species “of special interest” because they are most critical for biodiversity conservation. The desire of local people to protect their traditional community lands and livelihoods can be an effective biodiversity conservation strategy, which should be recognized in national biocultural policies.

**Supplementary Information:**

The online version contains supplementary material available at 10.1186/s13002-022-00567-z.

## Background

Mexico has been designated one of the world’s 12 megadiverse countries [1, 2]. Mexico is also a major centre of plant domestication [3]. Culturally, Mexico harbours an extraordinary number of Indigenous languages, by one estimate 364 languages of 64 major language families are spoken by over six million people [4, 5]. While such a concordance of biological and cultural diversity is clearly evident globally [6–9], the factors that may have favoured the coevolution of biological and cultural diversity are poorly understood and certainly complex. We believe that the linkage between biological and cultural diversity is as important for the future as it has been in the past.

A complex and deep historical relationship between the diversity of cultural expression and the diversity of life forms is particularly apparent in Mexico [10–12]. A good example is the diversity of Mexican languages and the diversity of Mexican birds—over 1100 species documented to date in Mexico [13]. Linguistic diversity has deep roots in time. Mexican language families such as Otomanguean, Mayan, and Yuto-Nahuan are estimated to have diversified in Mesoamerica over several millennia. The pre-Columbian fascination with birds is well documented in such colonial documents as the Florentine Codex (Sahagún). The poetry of Texcoco’s philosopher king, Netzahualcoyotl, the zoological collections of Moctezuma II, and the elaborate feather capes of the elite of Tenochtitlán also testify to the high cultural value ascribed to Mexico’s birds over the centuries [14–18]. Today, the descendants of these first Mexicans–and of the Spanish colonists since 1519–respect the spiritual power of many bird species and continue to value birds for what they contribute to local livelihoods, whether materially, aesthetically, or as signs and symbols of their ties to a homeland. We consider here how birds are appreciated in ten Indigenous Mexican communities. We compare how birds are named in the languages spoken in each of these communities and contrast local avian inventories with the diversity of local bird species recognized by ornithologists. We then note the varied roles these birds play in the daily life of each community. We compare the bird species accorded the most diverse and prominent cultural values in each, recognizing these as “high-value birds”. Some researchers have singled out one or more species as “cultural keystones” [19]. However, we find the concept of a keystone species to be problematic in this context. Given that birds are valued in so many different ways, reducing their value to a single indicator distracts from the complexity of the lessons these birds impart.

### Assessing the cultural value of birds

Cognition of the natural world has evolved in ways that permit humans to optimize the information required when making decisions about resource extraction and management. This is achieved partly through classification reflected in language [20]. Although not all features of the language are relevant to understanding how local people perceive and classify their environment, ethnobiological studies have developed in close proximity to linguistics, mainly because language is the point of access for studying and understanding local classification. If we are to understand the practical implications of ethnobiological classification, we must adopt a perspective that emphasises the complex and often fuzzy categories that people use rather than some abstract “natural” and unified scheme that might be inferred from some kinds of analysis [21].

Bird names are morpho-syntactically and semantically complex so familiarity with a local language is necessary to understand them. In fact, names with some “descriptive force” may reveal depths of local appreciation of the bird’s role in the local environment. Birds are also seen as a distinctive “natural” group of animals or intermediate categories suggesting that those groups are the primary organizing device in folk classifications using broad ecological and behavioural criteria that cannot easily be dismissed as “special purpose” [21–24].

Professional ornithologists classify birds at various levels in an ascending taxonomic hierarchy or “taxonomic tree.” Thus, a Chipping Sparrow (*Spizella passerina*) is also *Spizella*, as well as a “New World Sparrow”, in the family Passerellidae, and a “songbird” of the Passeriform order. In this system, the characteristics of importance are mainly morphological. However, according to the multidimensional classification model proposed by Alcántara-Salinas et al. [20, 21], indigenous people tend to classify birds using, as well as morphology, ecological, behavioural, and other characteristics in their day-to day life which clearly violate the integrity of so-called natural taxonomy.

For practical purposes, we count here only names for “terminal taxa”, that is those not further subdivided nomenclaturally. We count each name, whether it equates to a single Linnaean species or is applied more widely. We then consider the ratio between the professional ornithological avian inventory of species and the Indigenous inventory of bird names, which Hunn has defined elsewhere as the Scientific Species Recognition Ratio (SSRR)” [25]. The same idea is embodied in indices developed by other researchers. For example, Ellen [26, 27] calls it a “Differentiation or correspondence index”. This serves as our baseline for assessing the cultural value of birds in a particular community, but we need to ask to what extent the list of birds named locally approximates the comprehensive ornithological inventory for that same region. Table 1 shows some examples of the different language groups examined.

**Table 1:** Examples of local names for culturally significant birds for each language group studied, showing degree of correspondence between folk and scientific classification

Language	Local name	Latin name	English Name	Degree of correspondence	Number of species given same folk name
Cuicatec	*lúti indōho*	*Coragyps atratus*	Black Vulture	1:1	
	*‘iho kiáa*	*Ramphastos sulfuratus*	Keel-billed Toucan	1:1	
	*tíin dú*	Trochilidae		U	16 species
Northern Zapotec	*bërha rhiga*	*Ortalis vetula*	Plain Chachalaca	1:1	
	*bërha bke*	*Crax rubra*	Great Curassow	1:1	
	*chëbete*			U	84 species: for various species of small size birds or “dickey” birds
South Zapotec	*guiès-ró-yù*	*Leptotila verreauxi*	White-tipped Dove	1:1	
	*ngäs*	*Corvus corax*	Common Raven	1:1	
	*dzïng*			U	Trochilidae: 14 species
Tzeltal	*tuh kulum pukuh*	*Bubo virginianus*	Great Horned Owl	1:1	
	*čulin*	*Melanotis hypoleucus*	Blue-and-white Mockingbird	1:1	
	*šč’iht*			U	Most wood-warblers (Parulidae): 12 species
Maya	*jonkuuk*	*Harpia harpyja*	Harpy Eagle	1:1	
	k’ok’	*Turdus grayi*	Clay-coloured Thrush	1:1	
	takaay			U	Most flycatchers (Tyrannidae): at least 8 species
Nahuatl	*koxotl*	*Crypturellus cinnamomeus*	Thicket Tinamou	1:1	
	*tzatzi*	*Psilorhinus morio*	Brown Jay	1:1	
	*totoze kostik*			U	Most flycatchers (Tyrannidae in part): 13 species
Tlahuica	*nzaa mut’i*	*Buteo brachyurus*	Short-tailed Hawk	1:1	
	*chopaá*	*Toxostoma curvirostre*	Curve-billed Thrasher	1:1	
	*lindyuu*			U	Trochillidae: 7 species
Seri	*ac*	*Branta bernicla*	Brant	1:1	
	*xaláa*	*Campylorhynchus brunneicapillus*	Cactus Wren	1:1	
	*Coneenozíic*			U	Various sparrows (*Amphispiza* spp. and *Spizella* spp.)
Pima	*ñui*	*Cathartes aura*	Turkey Vulture	1:1	
	*shadam kakaichu*	*Callipepla squamata*	Scaled Quail	1:1	
	*bichpoḍ*			U	Abert’s and Canyon Towhees (*Melozone aberti, M. fusca*)
Kiliwa	*jajemiljamemil*	*Oreortyx pictus*	Mountain Quail	1:1	
	*tikuipai tayu*	*Gymnogyps californiaus*	California Condor	1:1	
	*pVnuet*			U	Swallows (Hirundinidae): 5 species

In column 5, 1:1 represents a one-to-one correspondence and U an under-differentiated category

In addition, there are bird names with “descriptive force”. Authors here registered “descriptive force” when the name of an animal was associated with a special symbolic value. For instance, in Cuicatec, *tsítu dōondi*, the Lesser Roadrunner (*Geococcyx velox*), is descriptive of sorcery, while *yódo ynohínhōo* is a “bird of the hot forest”, the Russet-crowned Motmot (*Momotus mexicanus*), is descriptive of its local range in the dry forest. In Northern Zapotec, solitaires (*Myadestes* spp.), *vigini artaba kia*, are “birds that ring bells”. In South Zapotec, *guiès-ró-yù*, the name of the White-tipped Dove (*Leptotila verreauxi*), is onomatopoeic, interpreted as “pot by the door”, while the name of the Bush tit (*Psaltriparus minimus*) *mziùud* < *rziùud*, “woven bag”, is descriptive of the nest. In Tzeltal, *tuh kulum pukuh,* the name of the Great Horned Owl (*Bubo virginianus*), is onomatopoeic, interpreted as “stinking devil”, while the name of the Yellow-eyed Junco (*Junco phaenotus*) is *tak’in sit mut,* “golden eye bird”. In Maya, the name of the Turkey Vulture (*Cathartes aura*) is *chak pool ch’oom,* “red-headed vulture,” and therefore, descriptive. In Nahuatl, the name of the “Blue-capped Motmot” and “Lesson's Motmot” *(Momotus coeruliceps* and *Momotus lessoni*), *kotlanhuehe*, is descriptive of the nest and viewed with respect as an “elder”. In Tlahuica, *lindyuu* (applied to several Trochilidae species) evokes certain religious practices. In Seri, the name *coneenozíic* > *conée ano ziic,* “bird in the grass”, describes the habitat of various grassland sparrows. In Pima, the name for the White-winged Dove (*Zenaida asiatica*), *o’okokoi* is onomatopoeic, evocative of its call, while the name of the Horned Lark (*Ermophila alpestris*), *ba’i chukul*, “black throat”, describes its colouration. Finally, in Kiliwa: *telpi,* the name of the Greater Roadrunner (*Geococcyx californianus*), is interpreted as “devil”.

These bird names demonstrate the intimate familiarity local people often have with the birds in their environment: a clear indication that the species are of cultural value. We begin our consideration of the cultural value of birds with the observation that they have names in the local language, plus the descriptive details often embodied in those names. We next consider other aspects of cultural value, whether material or non-material, whether conceptual, symbolic, or aesthetic.

Material value is directly relevant to local livelihoods. Birds may be hunted for food or for their skins, bones, or feathers. Domestic fowl, of course, also qualify, and in every Indigenous community studied, chickens (*Gallus gallus*), domestic turkeys (*Meleagris gallopavo*), and occasionally ducks (local types mainly derived from Mallards, *Anas platyrhynchos*, and Muscovy Ducks, *Cairina moschata*) were raised by local households. Though turkeys, Mallards, and Muscovy Ducks occur in the wild in Mexico, in our community sample they are most often known only as domesticates. A few species are considered to be medicinal (in the context of cultural diseases). In Tzeltal, *cokoy*, the Band-backed Wren (*Campylorhynchus zonatus*) is a medicine for warts, a treatment perhaps better considered under the heading “sympathetic magic” [28]. Likewise, the use of dried and powdered hummingbirds as a protective or love charm by the Maya is perhaps based on an appreciation of the power embodied in these tiny birds. Hummingbirds in Northern Zapotec, vultures among the Cuicatecs, and road runners among the Tlahuicas all have medicinal applications [29–31].

Symbolic values include stories and songs inspired by specific birds, birds that foretell good or bad fortune (augury or omen birds), birds that are associated with specific places (by which they may be named), birds that forecast weather patterns or seasonal changes, birds that mark specific habitats or specific connections with other animals or plants; in short anything reflecting the roles birds play in the mental life and conceptual practices of a community. According to Rea [32], “Almost all that is known to Western science about the behaviour and anatomy of [the turkey vulture] has somehow been encoded into Pima mythology… [no animal] looms so large in the Pima pantheon as Coyote and Buzzard [that is, the Turkey Vulture, *ñui*].” The Ancient Maya considered the Yellow-billed Cacique (*Amblycercus holosericeus*) a holy bird, named in the Book of Chilam Balam of Chumayel [33] and in Chunhuhub today this species is named simply “bird”, the bird *per excellence*. The Seri have composed many songs about birds.

To determine bird species of “special concern” for biodiversity conservation, we consulted “red lists” and lists of endemic species. Mexico has an established policy of protecting national biodiversity under SEMARNAT, which publishes lists of endangered species in the NOM-059-SEMARNAT-2010 [34]. It also follows international conventions, such as those of the IUCN [35], CITES [36], and BirdLife International, Partners in Flight Tri-National Vision for Landbird Conservation [37]. Mexico was a signatory to the Convention on Biological Diversity at the Rio Earth Summit (June 1992), through which it committed to protecting biological diversity and sustainable resource use. The Mexican government is also committed to the conservation of cultural diversity through the General Law on the Linguistic Rights of Indigenous People in Mexico [38] and through the National Institute of Indigenous Languages (INALI). It has ratified the Indigenous and Tribal Peoples Convention (ILO #169, 1989) and the Convention on the Protection and Promotion of the Diversity of Cultural Expression [39], as well as the UN Declaration on the Rights of Indigenous Peoples [40]. Finally, through the CBD [41] Mexico has affirmed that:

“Subject to national legislation, respect, preserve and maintain knowledge, innovations and practices of indigenous and local communities embodying traditional lifestyles relevant for the conservation and sustainable use of biological diversity and promote their wider application with the approval and involvement of the holders of such knowledge, innovations and practices and encourage the equitable sharing of the benefits arising from the utilization of such knowledge, innovations and practices” (Article 8j).

In the light of all these international agreements, one might conclude that biological diversity and cultural diversity are highly valued, globally and in Mexico in particular. However, the qualification “subject to national legislation” at the beginning of the CBD extract suggests that more needs to be done to coordinate efforts in defence of *biocultural diversity*, in Mexico and certainly elsewhere. We propose here concrete steps to integrate biocultural diversity conservation in Mexico. We use birds as an illustration, given that they are particularly prominent in the public imagination and are ubiquitous, conspicuous, and charismatic natural icons. As “canaries in a coal mine”, their welfare may serve as omens for humanity’s future, much as so many birds do for Indigenous observers. Formally recognizing the high-value birds of Mexico’s Indigenous communities should inspire *biocultural diversity* conservation as a national priority. We also examine the likelihood that a particular species of special concern will have been named—and how precisely named—in the local cultural inventory, to test the correlation between those birds most highly valued for biodiversity conservation and those of cultural value. If we find that cultural values and biodiversity values do not broadly overlap, we may nevertheless conclude that threatened species may still count on the protection of the local community using their land as a refuge.

## Methods

**Fig. 1:**
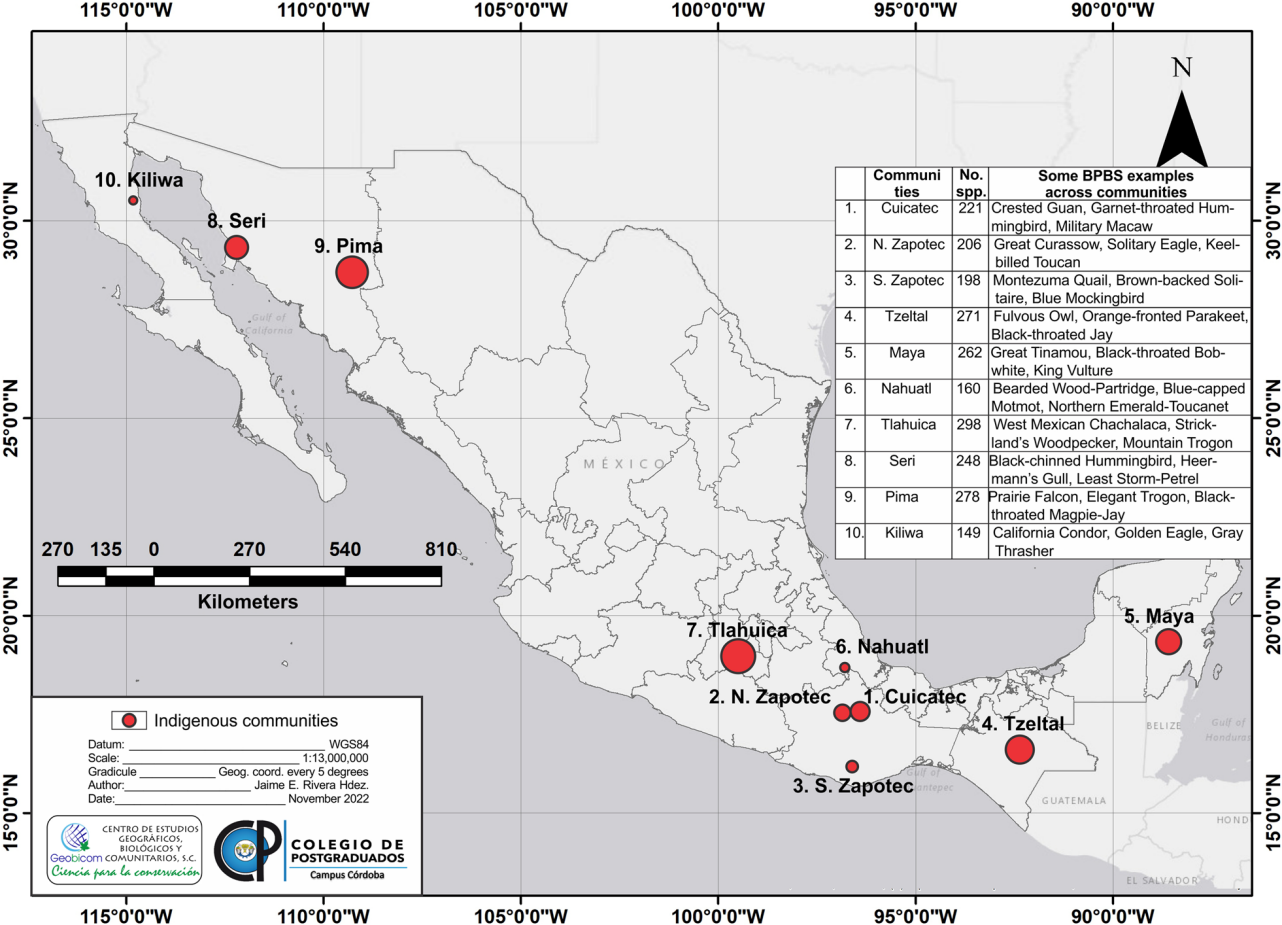
Map of indigenous communities located in red circles; the size of the circle simulates bird richness species; the additional table within the map shows the total of bird species with some examples of Biocultural Prominent Bird Species (BPBS) across communities

On the role of ethnoornithology and ethnobiology in biocultural conservation more generally see, for example, Hunn [42], Wyndham, Lepofsky and Tiffany [43], Wolverton et al. [44] and Tidemann and Gosler [45]. The ten ethnoornithological study sites (Fig. 1) referred to in this paper (Table 2) range along the full length of Mexico, from northern Baja California and the Sonoran-Arizona borderlands to Chiapas in the South and Quintana Roo in Yucatan Peninsula. The studies were conducted by researchers adopting different perspectives: ethnographic, linguistic, historical, and/or ecological. Thus, the data are not always easily compared. However, each represents an effort at a comprehensive account of local knowledge of those bird species likely to be encountered by community residents in the course of their daily lives.

**Table 2:** Descriptive data for study sites

Lang.	Loc.	Stt.	Lat. N	Len. W	Elev. (m.a.s.l.)	Pop.	Nm Tt	SSRR Tt/BS	Hab.
Cuicatec	San Juan Teponaxtla	Oaxaca	17° 40′	96° 45′	800–2800	730	99	0.45	COF, OF, CF, TEF, TDF, ASV, RWV
Northern Zapotec	San Miguel Tiltepec	Oaxaca	17° 30′	96° 25′	500–2600	369	102	0.50	COF, OF, CF, TEF, RWV
South Zapotec	San Juan Mixtepec	Oaxaca	16 ^o^49′	92° 30′	1600–3700	1000	104	0.53	XSG, COF, OF, TDF, RWV
Tzeltal	Tenejapa	Chiapas	16° 50′	92° 30′	900–2800	> 10,000	143	0.53	COF, OF, CF, TDF, ASV, RWV
Maya	Chunhuhub	Quintana Roo	19° 35′	88° 36′	10–50	6000	110	0.42	XSG, TEF, TDF, ASV, RWV
Nahuatl	Coetzapotitla	Veracruz	18 ^o^47′	96° 55′	620–1228	739	61	0.38	CF, TEF, RWV
Tlahuica	San Juan Atzingo-Loma de Teocaltzingo	Mexico State	19° 01′	99° 39′	2739	949	41	0.14	XSG, COF, OF, CF, ASV, RWV
Seri	Comcaac	Sonora	29° 50′	112° 39′	16	1000	120	0.48	XSG, TDF, ASV, RWV
Pima	Pima Bajo	Sonora/ Arizona	31° 52′	112° 75′	200–900	> 900	87	0.31	XSG, COF, OF, TDF, ASV, RWV
Kiliwa	Valle de Trinidad	Baja California	31° 40′	115° 73′	833	13	44	0.30	XSG, COF, OF, ASV

Lang. = language, Loc. = locality, Stt. = Mexico state, Lat. N = latitude, Len. W = length, Elev. (m.a.s.l.) = elevation (metres above sea level), Pop. = population, Nm Tt = number of the terminal taxa, SSRR = scientific species recognition ratio, Hab. = habitat sensu Rzedowski [64]: XSG = xerophilous scrub and grassland, COF = conifer forest, OF = oak forest, CF = cloud forest, TEF = tropical evergreen forest, TDF = tropical subdeciduous, deciduous and thorn forest, ASV = aquatic and subaquatic vegetation, RWV = ruderal and weed vegetation

The present authors were personally responsible for seven of the ten studies listed in Table 2. The seven studies followed the same procedure overall. First of all, we pursued the International Society of Ethnobiology Code Ethics to get the permission from local authorities and the community through a general assembly before starting the research. Bird data and ethnographic approaches were undertaken simultaneously, we were able to participate in most daily activities to gain rapport. We used participant observation as a default qualitative research strategy for long periods of fieldwork.

Both qualitative and quantitative data were subjected to systematization and constant analysis to get the ethnoornithological research with some variations here presented as follows: Alcántara-Salinas in San Miguel Tiltepec (Northern Zapotec) [29], San Juan Teponaxtla (Cuicatec) [30] and in Coetzapotitla (Nahuatl); Alcántara-Salinas with Ibáñez-Bravo in Arroyo León [46]; Alcántara-Salinas did fieldwork in San Juan Atzingo (Tlahuica) during 1997 and Aldasoro reported on San Juan Atzingo and La Loma de Teocaltzingo [31]. Alcántara-Salinas’s research was specifically and broadly ethnoornithological and in part linguistic. Hunn studied San Juan Mixtepec (South Zapotec) [47, 48] as part of a comprehensive ethnobiological study with a strong linguistic bias. He also studied Tenejapa (Tzeltal) [28] as part of a comprehensive ethnozoological study, again emphasizing nomenclature and classification.

We relied upon published accounts for three additional communities: Anderson’s Chunhuhub research (Quintana Roo, Yucatec Mayan) documented avian nomenclature and classification as part of an extensive ethnoecological study [33], while Rea’s long-term engagement with (Piman) natural history (especially Gila River Akimel O’odham, southern Arizona) produced a finely detailed catalogue of local knowledge and beliefs about birds, spanning the historic Piman range in Northern Mexico and the US borderlands. Rea in his book incorporated a range of historical documents [32]. The Seri, neighbours of the Northern Pima in Sonora State, were the focus of a comprehensive ethnoornithological monograph by Morales-Vera [49]. For pre-Columbian context, we consulted Navarijo-Ornelas [50].

The adequacy of our local bird lists, that is our assessment of each community’s “avian universe”, varies. Alcántara-Salinas, Hunn, Anderson, and Rea are all expert “birders.” They generated local bird inventories from personal observation over many years. Morales-Vera consulted detailed maps of avian ranges across Seri territory. Our bird inventories for San Juan Atzingo and Valle de Trinidad are based on the likely occurrence of species judged from maps in Howell and Webb’s field guide and probably exaggerate the local avifauna. These lists indicate the range of Mexican birds subject to community oversight and traditional conservation practices. Most fall between 200 and 270 species, averaging 229. Taken together these ten communities host 769 species, 69% of the total Mexican avifauna. If we exclude oceanic species, island endemics, vagrants, and extirpated species, the Mexican list is reduced to 938. Of these, our collective Indigenous community “avian universe” includes 82% of the total.

With respect to species given “biological conservation status” for biodiversity conservation, we have counted the total number of Mexico’s birds listed in one or more of the five published “red lists” described above. Of Mexico’s 1115 documented bird species, 262 are listed as being of “special concern”, 21% of the total. We have counted how many of these species of special concern occur within the “avian universes” of each of our ten Indigenous communities and the fraction of the total lists for each. These numbers are provided in Table 3.

**Table 3:** Relationship between bird species universe, bird species of concern for each locality, and area

1	MEXICO	CUI	NZP	SZP	TZE	MAY	NAH	TLH	SER	PIM	KIL	AVERAGE
2	1115	198	221	206	271	262	160	298	248	278	149	229.1
3	262	70	87	77	74	79	49	75	32	46	27	61.6
4	0.23	0.35	0.39	0.37	0.27	0.30	0.31	0.25	0.13	0.17	0.18	0.27

Numbers in the first column mean: (1) region; (2) number of species in the “avian universe” of that region; (3) number of “species of special concern” in that region; and (4) fraction of the total regional species that are of “special concern”

CUI = Cuicatec, NZP = Northern Zapotec, SZP = South Zapotec, TZE = Tzeltal, MAY = Maya, NAH = Nahuatl, TLH = Tlahuica, SER = Seri, PIM = Pima, KIL = Kiliwa

Seven of the ten study sites harbour a large percentage of species of special concern for biodiversity conservation compared with Mexico as a whole. A gradient from south to north in the proportion of such species in each community likely reflects the distribution of Mexican endemic species as well as the differential concentration of the most unique habitats across these communities.

We also consider the correlation between local species of “high biological conservation status or value” with those of “high cultural value”. Cultural value first of all depends on cultural recognition, which we argue requires some level of naming in the local vernacular, whether in an Indigenous language or in local Spanish. Indigenous language names remain current in half of our community sample: the two Zapotec communities in Oaxaca, the two Mayan communities in Chiapas and Quintana Roo, and the Seri locality in Sonora. Northern Pima vocabulary is preserved in the memories of a few elders and in historic sources. In the other communities, the Indigenous language nomenclature is likely a pale reflection of the aboriginal detail.

Other indices of cultural value, whether material or symbolic, differ dramatically among our communities. The Pima and Seri communities in northern Mexico exhibit extensive engagement with local birds in song and story as well as prey of local hunters. Other communities seem impoverished by comparison. It is perhaps not surprising that this contrast correlates with the northern communities’ greater reliance on hunting and gathering rather than intensive agriculture for their basic subsistence.

### Ethnographic documentation

We have summarized descriptive data for the 10 study sites involved in Table 2. Here, we provide some other relevant data on ethnographic characteristics.

1. San Juan Teponaxtla, Oaxaca, Cuicatec (Otomangue language) is located between the Sierra Norte and Cañada regions inhabited by both mestizo and Indigenous people, for whom the principal source of subsistence is agriculture. Since the opening of the road connecting Teponaxtla to Cuicatlán, people sell products such as fruits and vegetables to boost household income. The ethnoornithological data were recorded by Graciela Alcántara-Salinas, Diego-Alexandro Rivera-Alcántara and Jaime E. Rivera-Hernández from summer 2007 to summer 2008 as part of a doctoral dissertation. The research involved a comparative ethnoornithological study of Zapotec and Cuicatec communities in Oaxaca on bird ethnoclassification and nomenclature, uses, symbolism, and a bird inventory [21, 30].
2. San Miguel Tiltepec, Oaxaca, Northern Zapotec (Otomangue language) is located in the Sierra Norte, and comprises Zapotec and Spanish-speaking Indigenous people. Their productive activities are characteristic of local self-sufficient peasant agricultural economies based on maize, chillies, beans, and squashes, plus gathering wild plants and occasionally hunting wild animals. The ethnoornithological data were documented by Graciela Alcántara Salinas, Donato Acuca Vázquez, and Ausencia López Cruz as part of a Master of Science dissertation from the UNAM from 1997 to 1998 and 2000 and authorized by the local authorities and by the community assembly. The research involved recording Zapotec nomenclature for birds and bird anatomy and ethnographic documentation of uses, symbolism, and a comprehensive bird inventory [20, 29, 30, 51].
3. San Juan Mixtepec, Oaxaca, Cisyautepecan, South Zapotec (Otomangue language). This settlement lies in the rain shadow of the Sierra de Miahuatlán and has been an independent Zapotec-speaking community on its present territory since before the Spanish conquest. Residents today depend primarily on subsistence agriculture. The ethnoornithological data were recorded by Eugene Hunn and Donato Acuca Vásquez in 1996–1998 as part of ethnobiological research in the site area, duly authorized by the community. A general ethnographic account is published in Hunn [47] (with a digital archive at faculty.washington.edu/hunn/zapotec/), while the details of local biological classification and nomenclature are summarized in Hunn [25]. A comprehensive list of 191 bird species identified in the immediate vicinity of this community can be found in Hunn, Acuca Vásquez, and Escalante [48].
4. Tenejapa, Chiapas Tzeltal (Mayan Language). On the Atlantic slope of the Central Highlands, this has been an autonomous Mayan-speaking community since before the Spanish conquest. Tzeltal remains the dominant local language. The ethnoornithological data were recorded by Eugene Hunn in 1971 and 1972 for his PhD dissertation at the University of California, Berkeley. Hunn [28] visited each *paraje* of the *municipio* of Tenejapa, as well as neighbouring communities, recording each species of bird encountered, most often in the company of bilingual (Tzeltal/Spanish) Tenejapan guides. The local consultants provided bird names and commentaries on the cultural significance of the species encountered. He interviewed residents, including at least one from each of the 25 named communities within Tenejapa, to obtain a representative sample of local zoological knowledge. Mist nets were used to capture birds for more positive identification. He recorded 217 species of birds in the Central Highland region of Chiapas, within the life range of Tenejapa.
5. Chunhuhub, Quintana Roo, Yucatec (Mayan Language). This location on the Yucatán Peninsula was resettled beginning in the 1940s by Maya displaced by the Caste Wars of the mid-1800s. The ethnoornithological data were recorded by E. N. Anderson with the active collaboration of his local colleague Félix Medina Tzuc during several intervals between 1991 and 2001 [33]. Anderson’s primary focus in this research was on issues of political ecology, with classification and nomenclature as a secondary focus.
6. San Juan Atzingo and Loma de Teocaltzingo, State of Mexico, Tlahuica (Otomangue language). In 1996 from March to October, Alcántara-Salinas undertook ethnoornithological fieldwork in San Juan Atzingo, obtaining a bird checklist of 79 species and Tlahuica bird nomenclature. Aldasoro conducted ethnobiological research through participative action research from 2007 to 2012 in San Juan Atzingo and La Loma de Teocaltzingo. In collaboration particularly with an ecotourism project, they together registered 59 folk taxa corresponding to 58 species, 29 Families and 13 orders. 66 names in Spanish and 53 in Tlahuica were also documented, as well as the uses, practices and beliefs associated with these birds.
7. Coetzapotitla, Veracruz, Nahuatl (Uto-Aztecan language). The main productive activity of these Spanish and Nahuatl speakers of the Sierra de Zongolica is farming maize, beans, tepejilote (*Chamaedorea tepejilote*), and velillo (banana leaf). Ethnoornithological data were obtained by Graciela Alcántara-Salinas and Jaime E. Rivera-Hernández starting in 2018 and since 2020 with Antonio Pérez-Sato, Natalia Real-Luna and Rafael Muñoz-Márquez, as part of an ecotourism project for the Master’s Program on Rural Tourism and Landscape at the Postgraduate College in Córdoba. Results have not yet been published.
8. “Rancherias”, Sonora, Northern Piman (Uto-Aztecan language). This study was principally undertaken at the Gila River Indian Reservation, Arizona, USA, but with comparative material from Tohono O’odham and from Northern Piman in Sonora, Mexico. The local environment combines the Sonoran Desert and riparian habitats. Northern Piman today live on three reservations in Arizona and at scattered *rancherías* in Sonora. They continue to harvest the traditional wild plant and animal foods as well as farming on the margins of the remnants of rivers and flooded stream that cross their traditional territory. The ethnoornithological data were recorded by Amadeo Rea between 1963 and 2007 [32, 52], over a lifetime of close association with various Northern Piman friends. Rea has very carefully documented cultural beliefs and practices related to birds, the primary focus of his research.
9. El Desemboque, Sonora, Seri (Independent language), Sonora, Mexico. This study concentrated on two settlements, Punta Chueca and El Desemboque, both Seri and Spanish speakers. Their traditional subsistence was hunting and gathering, including the harvesting of resident sea turtles. Their territory extends from Puerto Libertad in the north to Bahía de Kino in the south, to include Isla Tiburón and the Canal del Infiernillo. According to Morales-Vera, these locations comprise a Natural protected area. In total, they occupy 2110 km^2^, 910 km^2^ in *ejidos*, and 2100 km^2^ of communal land. The habitat is low elevation Sonoran Desert, the vegetation classified as “Matorral xerófilo (xerophytic scrub)”, “Matorral desértico micrófilo (microphyllous desert scrub)” and “Manglar (mangroves)”. We have been able to benefit from a detailed ethnoornithological monograph by Morales-Vera [49]. This report is richly annotated with Indigenous stories and songs inspired by the local birds. Complementing Morales’ ethnoornithology is the detailed ethnoherpetological account of Nabhan [53] and the ethnobotanical account of Felger and Moser [54].
10. Valle de la Trinidad, Baja California, Kiliwa (Yuman language). The ethnoornithological data for these Kiliwa and Spanish speakers were obtained by Elena Ibáñez, Maribel Alvarado, Jorge Arroyo, Eva Caccavari and Graciela Alcántara-Salinas with the support of José Ochurte Espinoza, one of the last speakers in this language. Fieldwork was carried out from 2014 to 2016 and it has been part of the INALI project for language documentation. The project has produced didactic materials including ethnoornithology [46, 55]. Kiliwa is a language at risk of extinction [56, 57]. According to Mixco [58], Kiliwa is a culture of hunter-gathering tradition and clan organization, and he describes it as a culture of the “Archaic desert”. They are located in the ejido of Kiliwa, previously known as Arroyo de León, in the municipality of Ensenada, Baja California. Their traditional territory previously covered the area from San Felipe Bay to the mountains where the San Pedro Mártir National Park is located. Currently, there are just between five and six speakers with different degrees of bilingualism identified.

### Bird list analysis

In order to analyse taxonomic composition in each study area, we counted species, families, and orders, and for each we calculated the proportion of the total. The status and distribution of species follow Howell and Webb [59]. These are: R = resident (breeds and resides within its range throughout the year); SR = summer resident (breeds in the region but is present only for a period during the northern summer); W = winter visitor (non-breeding visitor present during the northern winter); T = transient (non-breeding visitor only present during spring and/or autumn migration); plus END = all the endemic and quasi-endemic species determined according to Binford [60], Howell and Webb [59] and Berlanga et al. [13].

### Correspondence analysis

We ran a correspondence analysis which is a method of data analysis to represent in a graph tabular data in order to maximize the correlation of many variables involved, giving rise to a principal axis and scores. Axis I and II always have the largest eigenvalues (Additional file 1: Appendix I) that explain the largest variance within the data. From Axis III onwards, the explanation of the analysis is just residual variation and successively smaller eigenvalues [61, 62].

The correspondence analysis used here allows us to relate bird cultural value to bird biological conservation status. We considered bird species as being of “special conservation [biological] status” when listed in the following conservation policy documents: (1) International (IUCN, CITES, VV, USFWS), (2) National (NOM-059-SEMARNAT-2010), and (3) END (Endemic). We also considered the following cultural values: (4) NR (nomenclatural recognition), (5) material uses (as food, medicine, in sorcery, as ornaments, and in religious offerings), (6) symbolic use (omens, oral tradition), and (7) ecological value for each species from the point of view of the local communities studied. We constructed a matrix for 1275 bird species using these seven indicators. The correspondence analysis was run using InfoStat/L-v2020 software [63]. The number of species (1275) used for this analysis included species repeated across the 10 research areas.

## Results and discussion

For the ten communities studied here, a total of 769 (Additional file 2: Appendix II) bird species were reported in or near each communal territory, representing 24 orders and 89 families. These occur in approximately one per cent of the Mexican national territory within nine of the 12 types of vegetation described by Rzedowski [64] as follows: XSG = xerophilous scrub and grassland, COF = conifer forest, OF = oak forest, CF = cloud forest, TEF = tropical evergreen forest, TDF = tropical subdeciduous, deciduous, and thorn forest, ASV = aquatic and subaquatic vegetation, RWV = ruderal and weed vegetation.

According to Mexican law (NOM-059-SEMARNAT-2010), 165 bird species have official bird conservation status [34], whereas the International Union for Conservation of Nature lists 50 species as of special concern for conservation [35]. Other International conservation guidelines we considered were VV of *The Partners in Flight ACAD Book* [37] and the birds of conservation concern in the USFWS [65], which listed 378 and 148 species, respectively.

**Fig. 2:**
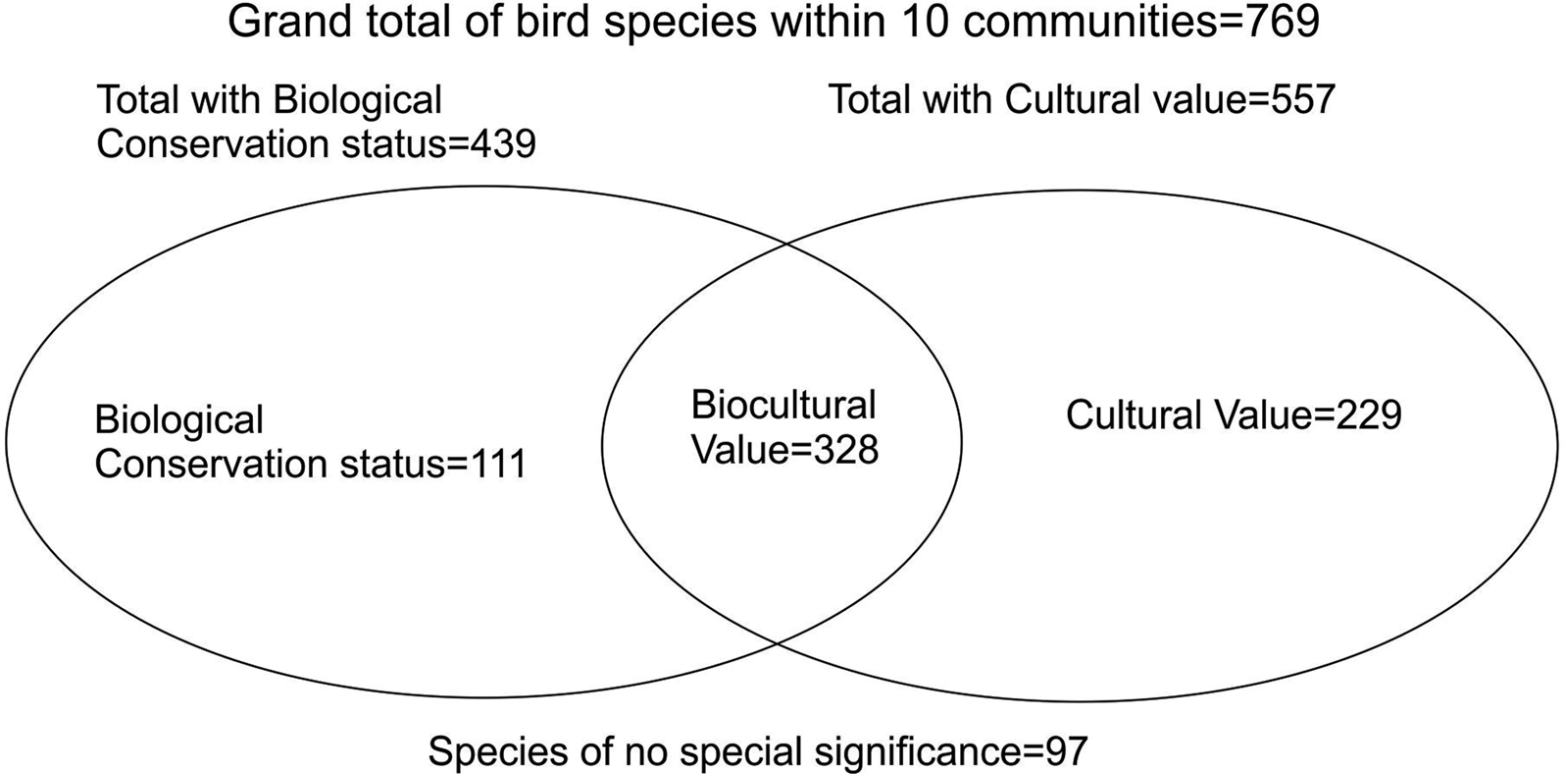
Venn diagram showing total bird species in 10 Mexican Indigenous territories listed as being of biological, cultural, and biocultural value according to definitions used in this study

Of the 769 species present in our 10 Indigenous study sites, 122 are endemic, quasi-endemic, or semi-endemic; 429 are resident; 125 are winter migrants; five are summer migrants; 12 are transitory; five are accidental; and 193 have more than one distribution status. Of the same 769 species, 557 were of some cultural relevance, 439 were considered to be of special bird conservation status; and 678 were noted as of either cultural significance, conservation concern, or both. Of these, 328 species were noted for both cultural and biological value; 229 species had cultural value but lacked special biological concern, while 111 were rated as of special biological concern but were not mentioned for cultural value. Finally, 99 species presumed to occur within the life space of our 10 Mexican indigenous communities lacked cultural or biological significance (Fig. 2).

**Fig. 3:**
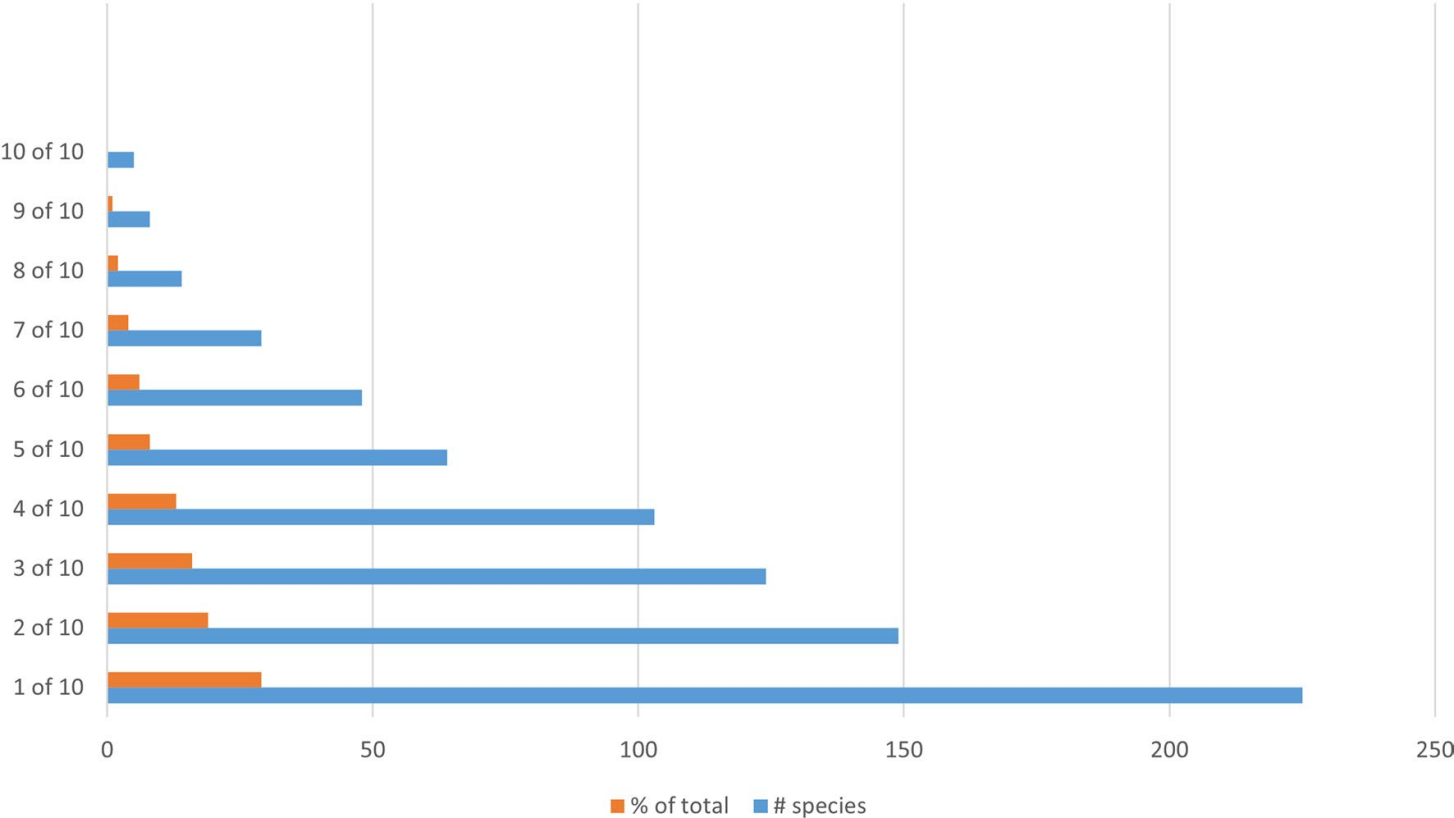
Distribution of local species across communities

Of the total of 769 species recorded in the whole study areas, 225 were listed in just one community, 149 in two, 124 in three, 103 in four, 64 in five, 48 in six, 29 in seven, 14 in eight, 8 in nine, and 5 of the whole communities (Fig. 3).

**Fig. 4:**
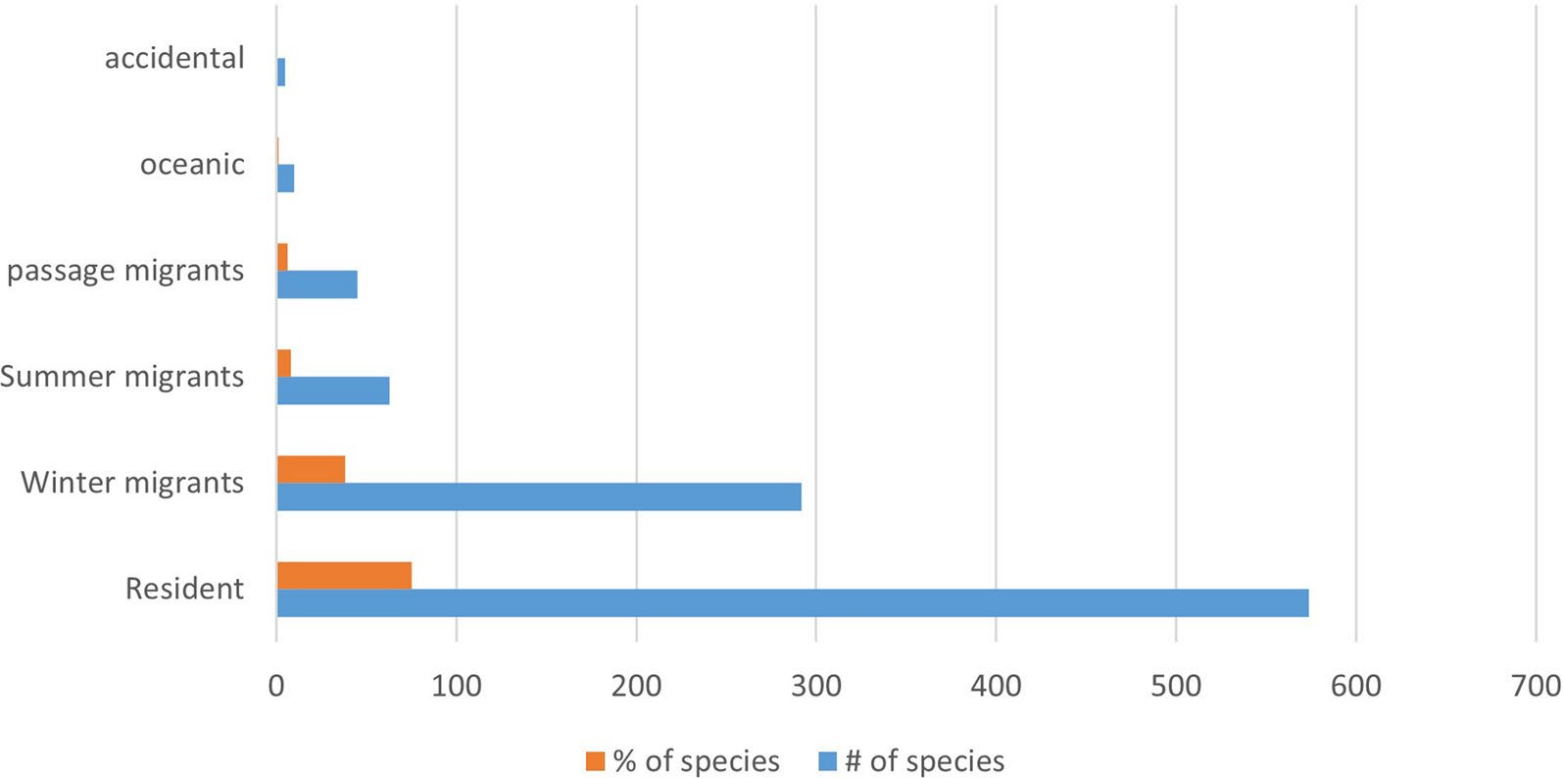
Distribution of species according to seasonal status

The most frequently noted species included domesticates and species that are both widespread and notable. The least frequently noted are species of specialized habitats, notably marine species that feature in the avian universe of just one or a few communities, such as the Seri. Of the 769 species listed, 574 were permanent residents (though some, 145, also had migratory populations); 292 species were (partly or wholly) migratory, wintering in Mexico (breeding to the north), with 63 species migrating to Mexico to breed in summer (though some of these might also have resident and/or wintering populations); 45 were “transients” (though a few of these could also be resident or migrants); 10 were “oceanic” (mostly recognized by the Seri in Sonora); and 5 were considered “accidental”, that is, not of regular occurrence in Mexico (Fig. 4).

**Fig. 5:**
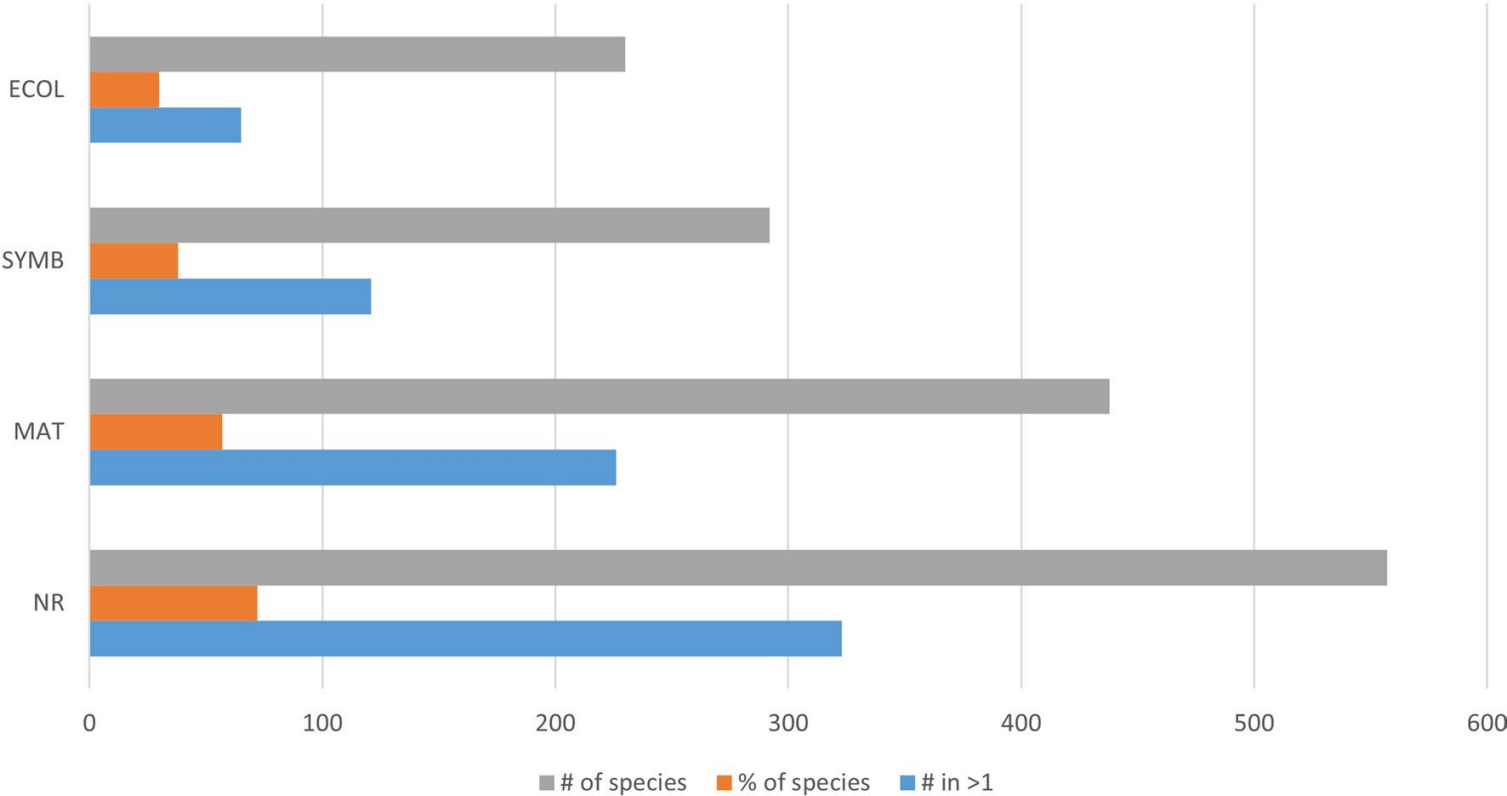
Distribution of cultural values

Of the 769 species under consideration, 435 had some “bird conservation status” (that is were listed on one or another list of species of special conservation concern). These included 382 on international lists and 251 on Mexican National lists; 237 species were listed on either the International or the National lists, but not both; 198 were listed on both International and National lists. With regard to “cultural values”, 557 were named (NR) in at least one community; of these, 323 were named in two or more communities; 438 species were cited for “material values” (MAT) by at least one community, while 226 of these were cited by two or more communities; “symbolic values” (SYMB) were noted for 292 species in at least one community, of which 121 were noted for their symbolic value in two or more communities; with respect to “ecological indicator values” (ECOL), 230 species were so valued in at least one community, with 65 of those so noted in two or more communities (Fig. 5).

### Correspondence analysis

We applied a correspondence analysis to these data which indicated that two main axes explained 81.55% of the variation. This demonstrates a strong relationship (*p* < 0.0001) between the 10 bird species within the communities, bird conservation status, and cultural value.

**Fig. 6:**
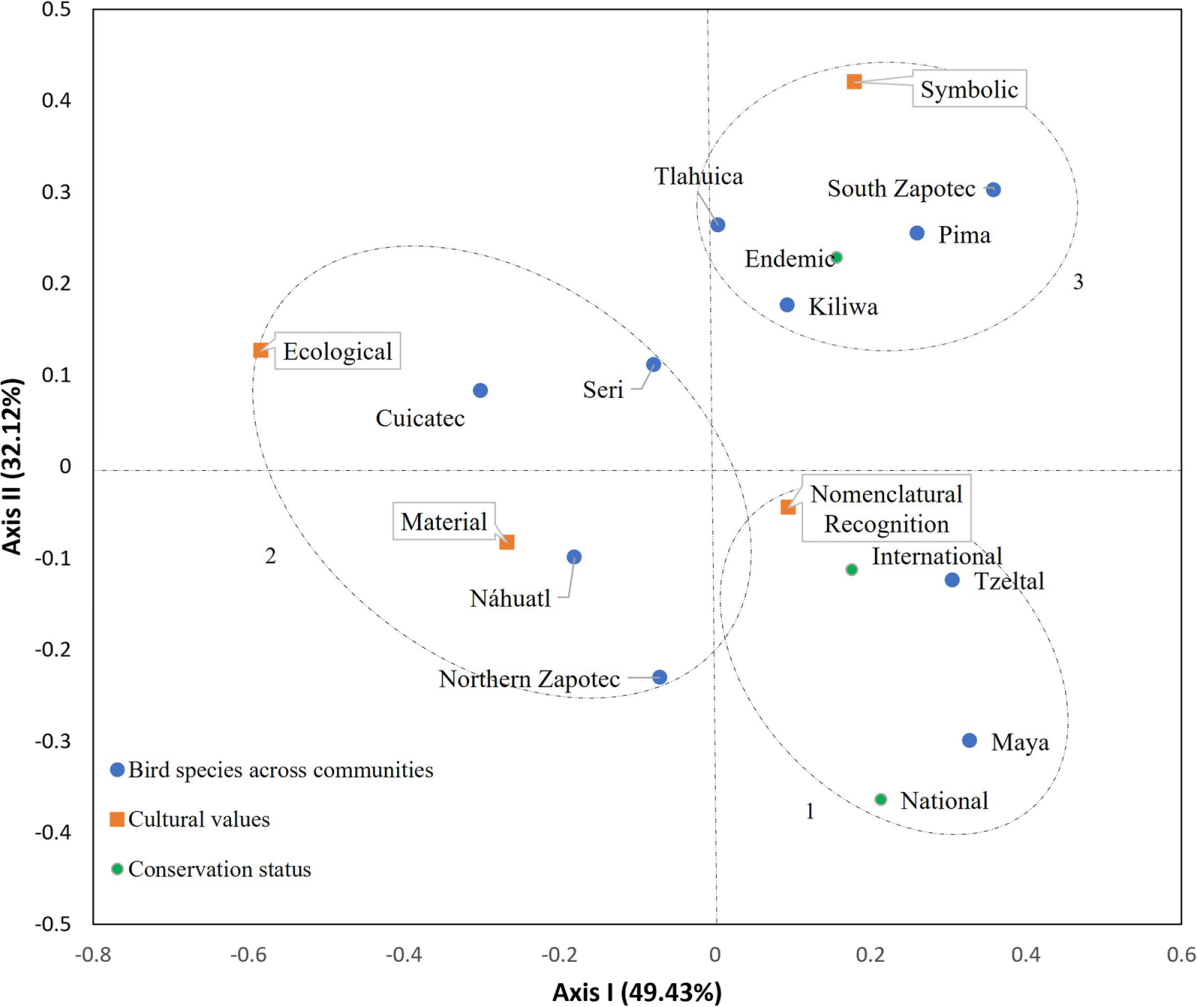
Correspondence analysis of biocultural data by community. The graph shows the correlation of variables: bird species by community, cultural value, and their conservation status. Axis I and II always have the largest eigenvalues explaining the overall analysis

In examining Fig. 6, we can see the 10 indigenous communities and the correlation between bird cultural values and conservation status, the relationship among them is indicated within a delineated ellipse conforming to three main groupings. Group 1 birds of the Maya (0.328, − 0.299) and Tzeltal (0.305, − 0.123) communities with the species that maintain a conservation status for both national (0.213, − 0.363) and international (0.176, − 0.112) threatened lists, as well as by their nomenclatural recognition (0.094, − 0.045). In group 2, there is a strong relation between ecological (− 0.583, 0.126) and material (− 0.267, − 0.083) cultural values of bird species with the communities of Northern Zapotec (− 0.070, − 0.230), Cuicatec (− 0.301, 0.082), Seri (− 0.078, 0.111), and Nahuatl (− 0.180, − 0.099). Group 3 brings together birds of the Tlahuica (0.004, 0.263), Pima (0.260, 0.254), South Zapotec (0.358, 0.302), and Kiliwa (0.093) communities connecting their endemic status (0.156, 0.228) to their symbolic (0.179, 0.419) use.

It is worth mentioning that all three groups formed in the correspondence analysis are determined by the cultural values that each community gives to its birds. There is also an important variation in the number of species with some conservation status in National and International lists. The fact that there are 435 species with some conservation status for the 769 registered in the ten localities implies that the communities do not have the same number of species with conservation status as are culturally valued. However, the global analysis of correspondence with the explained variation of 81.55% is a high value that shows the close relationship between the conservation value of bird species and the cultural values that each community gives them.

### Relevance to public policy

As we have shown through this analysis, cultural values are strongly correlated with the perceptions of professional biologists regarding the extent to which bird species for each locality are threatened. We hope that documenting a strong correlation between scientific and local perceptions of significance will help motivate public policy to protect “Bioculturally Prominent Bird Species” (hereafter BPBS) and to move towards the conservation of species of special biocultural importance, not only birds but other biological groups as well. Here, we propose three possible categories of BPBS relevant to public policy as follows:

#### High BCPBS:

Those species are used by indigenous people in a multiplicity of ways and are involved in a wide range of social and cultural practices, both material and non-material. These include those appearing on any threatened list, but also those considered endemic. Examples are: West Mexican Chachalaca (*Ortalis poliocephala*), Long-tailed Wood-Partridge (*Dendrortyx macroura*), Banded Quail (*Philortyx fasciatus*), Blue-throated Mountain-gem (*Lampornis clemenciae*)*,* Strickland's Woodpecker (*Dryobates stricklandi*)*,* Gray-barred Wren (*Campylorhynchus megalopterus*), Bearded Wood-Partridge (*Dendrortyx barbatus*), Bumblebee Hummingbird (*Selasphorus heloisa*), among others.

#### Medium BCPBS:

Those species used by indigenous people in a multiplicity of ways, and species involved in a wide range of social and cultural practices, both material and non-material. These include those appearing on any threatened list, but not endemic. Examples are: Northern Emerald-Toucanet (*Aulacorhynchus prasinus*), Chestnut-coloured Woodpecker (*Celeus castaneus*), White-crowned Parrot (*Pionus senilis*), Brown-backed Solitaire (*Myadestes occidentalis*), Slaty-breasted Tinamou (*Crypturellus boucardi*)*,* Spotted Wood-Quail (*Odontophorus guttatus*)*,* Solitary Eagle (*Buteogallus solitarius*), Collared Forest-Falcon (*Micrastur semitorquatus*), among others.

#### BCPBS:

Species not necessarily listed on any threat list, but with a wide range of social and cultural uses, both material and non-material. Some examples are: Turkey Vulture (*Cathartes aura*), Barn Owl (*Tyto alba*), Gartered Trogon (*Trogon caligatus*), Green Kingfisher (*Chloroceryle americana*), White-tipped Dove (*Leptotila verreauxi*), Killdeer (*Charadrius vociferus*), Great Horned Owl (*Bubo virginianus*), and Northern Beardless-Tyrannulet (*Camptostoma imberbe*).

It is necessary to first instantiate public policy regarding BPBS at the local level, and then move to regional and state levels, given the high biological and cultural richness throughout the country. One significant problem in Mexico when considering the biocultural conservation of species is the existing lack of public policy, especially given that this is one of the most bioculturally diverse countries in the world. Other problems have been the influence of a history of neoliberal economics on land use in rural and Indigenous communities, which has increased poverty and material inequalities, and led to overall social polarization, over-extraction of natural resources and a high degree of social marginalization. All these factors have contributed to a “cascade effect”, not only in terms of biodiversity depletion but also in terms of social and cultural deprivation. Prohibition of Indigenous languages throughout Mexico from the nineteenth century onwards, when ethnic and linguistic identities were systematically suppressed and devalued, coupled with urban and international labour migration, religious change, new forms of education, and social benefit programmes (“Progresa”, “Oportunidades” and “Prospera”) reinforced by neoliberalism, are among the main sociocultural and economic changes that have impacted negatively on traditional knowledge. We can see this, for example, among Zapotec and Cuicatec indigenous groups in Oaxaca [30].

The erosion and loss of traditional knowledge are largely associated with a breakdown in the mechanisms for its transmission. This is linked to a reduction in the extraction of natural resources through the loss of adult male workers to migration, and little engagement in local commercial development. The social benefit programmes may create dependence on external products and discourage local production. Although the current government tries to support the cultural values of ethnic groups, a biocultural vision is strongly required. Environmental policy has been neglected. Not only does Mexico need economic support for social minorities, but it is necessary to ensure the conservation of Mexico’s natural capital and to reduce budget cuts in CONABIO, CONANP, PROFEPA, SEMARNAT, among other supportive organizations. For these reasons, there is an urgent need to demonstrate the importance of bioculturally informed conservation strategies that can rapidly and accurately assess rates of biodiversity loss in relation to the retention of traditional knowledge for different age and gender groups. This will allow us to understand how biological loss impacts traditional knowledge and therefore cultural diversity and vice versa. Important also is the impact of illegal wildlife trade. The Indigenous communities of Oaxaca are mostly reliant on self-consumption, and cultural codes generally specify the upper limits for both numbers of hunters and animals caught, but where such codes have been undermined and outcomes determined by the demand for protected species hunting become unsustainable [30]. This is another reason why we need to stress the importance of biocultural conservation. The research we carry out in Indigenous and rural communities requires a holistic approach where the local actors are considered not as objects of public policy and Mexican law, but active agents on whom ultimately depends the survival of biocultural diversity [31]. We propose the term Bioculturally Prominent Bird Species to suggest a new way of conceptualizing the relationship between humans and wider nature, which will have positive consequences for biocultural conservation. This concept is consistent with the recommendation of others to establish Biocultural Communitarian Protocols (BCP). BCPs are charters with rules and responsibilities, in which communities establish their customary rights over their natural resources and territory, according to customary, national and international laws [66]. But the concept also highlights the role of ethnobiologist in conservation policies as proposed by Hunn [42], Wyndham [43] Wolverton, Nolan and Ahmed [44] and Tidemann and Gosler [45]

## Conclusions

Biodiversity conservation is typically based on a methodology that identifies biological taxa in need of protection through internationally agreed “objective (scientific) criteria”, that are assumed to be valid cross-culturally and cross-nationally, irrespective of ecological or sociocultural differences. In practice, however, the value people attribute to a species also reflects local cultural perceptions and uses, although in conservation contexts these values are either suppressed, ignored, or taken for granted. We argue here that conservation needs to adopt a biocultural approach that takes into account not only international scientific agendas, but also the values and priorities of local people, to move from “top-down” to “bottom-up” initiatives, that recognize the role of “local” and “regional” experiences that include use, management, and worldview to build biocultural strategies for more effective conservation.

## Supplementary Information

**Additional file 1: Appendix I.** Eigen values for each main Axis in the correspondence analysis by locality.

**Additional file 2: Appendix II.** Check List of bird species and their biological and cultural values for each study site.

## Data Availability

All data generated or analysed during this study are included in this manuscript as Additional file 1: Appendix I and Additional file 2: Appendix II.

## Abbreviations

CBD: UN Convention on Biological Diversity
CITES: The Convention on International Trade in Endangered Species of Wild Fauna and Flora
CONABIO: National Commission for Knowledge and Use of the Biodiversity
CONACYT: National Council of Science and Technology
CONANP: National Commission of Natural Protected Areas
INALI: The National Institute of Indigenous Languages
INEGI: The National Institute of Statistics and Geography
INFOSTAT: A general-purpose software for statistical analysis using Window
IUCN: International Union for Conservation of Nature
GEOBICOM S.C.: The Centre of Geographic, Biologic and Community Studies, Civil Society
NOM-059: Mexican Official Decree number 059
PROFEPA: Federal Attorney of Environment Protection
SERBO A.C.: The Society for the Study of Biotic Resources of Oaxaca, Civil Association
SEMARNAT: Secretariat of Environment and Natural Resources
UNAM: National Autonomous University of Mexico
USFWS: US Fish & Wildlife Service
VV: Vulnerability Value
