# Supplementary material for: Bird conservation status and cultural values in Indigenous Mexican communities: towards a bioculturally informed conservation policy
Source: J Ethnobiol Ethnomed. 2022 Dec 2;18:69. doi: 10.1186/s13002-022-00567-z (PMC9719214; doi:10.1186/s13002-022-00567-z)
Supplement: Supplementary file 1 — Additional file 1: Appendix I. Eigen values for each main Axis in the correspondence analysis by locality. [file 13002_2022_567_MOESM1_ESM.docx]

**Appendix I.**

Eigen values for each main Axis in the correspondence analysis by locality. Highest values are marked *

| **Communities** | **Axis 1** | **Axis 2** |
| --- | --- | --- |
| Cuicatec |  |  |
| Kiliwa | 0.0931 | 0.1761 |
| Maya | 0.3280* | -0.2997* |
| Nahuatl | -0.1803 | -0.0991 |
| Northern Zapotec | -0.0702 | -0.2305 |
| Pima | 0.2601 | 0.2546 |
| Seri | -0.0781 | 0.1111 |
| South Zapotec | 0.3588* | 0.3020* |
| Tlahuica | 0.0043 | 0.2634 |
| Tzeltal | 0.3055 | -0.1238 |

Eigen values obtained for each main Axis in the correspondence analysis per biocultural values. Highest values are marked *

|  | **Axis 1** | **Axis 2** |
| --- | --- | --- |
| Ecological | -0.5836* | 0.1266 |
| Endemic | 0.1564 | 0.2284 |
| International | 0.1760 | -0.1124 |
| Material | -0.2673* | -0.0831 |
| National | 0.2130 | -0.3637* |
| Nomenclatural recognition | 0.0946 | -0.0452 |
| Symbolic | 0.1796 | 0.4198* |
