# Supplementary material for: Bird conservation status and cultural values in Indigenous Mexican communities: towards a bioculturally informed conservation policy
Source: J Ethnobiol Ethnomed. 2022 Dec 2;18:69. doi: 10.1186/s13002-022-00567-z (PMC9719214; doi:10.1186/s13002-022-00567-z)
Supplement: Supplementary file 2 — Additional file 2: Appendix II. Check List of bird species and their biological and cultural values for each study site. [file 13002_2022_567_MOESM2_ESM.docx]

**Appendix II.** Check List of bird species and their biological and cultural values for each study site.

The first column corresponds to the study site number where each species was recorded: 1. Kiliwa, 2. Pima, 3. Seri, 4. Tlahuica, 5. Nahuatl, 6. Northern Zapotec, 7. South Zapotec, 8. Cuicatec, 9. Tzeltal, 10. Maya. NAT= Mexican law (NOM-059-SEMARNAT-2010) and Endemism, INT= IUCN, VV and USFWS, NR= Nomenclatural Recognition (X), MAT= Material Use (edible, ornamental, medicinal, etc.), SYM= Omen, ECO= Ecological value for people (X), DIS= R-Resident, MI-Winter visitor, MV-Summer visitor, T-Transient, O-Oceanic, A-Accidental. For each cell, X-means recorded in one of the ten study sites, M-means recorded in multiple study sites

| **Community** | **Taxonomic categories** | **NAT** | **INT** | **NR** | **MAT** | **SYM** | **ECO** | **DIS** |
| --- | --- | --- | --- | --- | --- | --- | --- | --- |
|  | **Tinamiformes** |  |  |  |  |  |  |  |
|  | **Tinamidae** |  |  |  |  |  |  |  |
| 6, 10 | *Tinamus major* | X | X | M | X | X |  | R |
| 10 | *Crypturellus soui* | X |  | X |  |  |  | R |
| 5, 9, 10 | *Crypturellus cinnamomeus* | X | X | M | M | X |  | R |
| 6 | *Crypturellus boucardi* | X | X | X | X | X |  | R |
|  | **Anseriformes** |  |  |  |  |  |  |  |
|  | **Anatidae** |  |  |  |  |  |  |  |
| 3, 7, 9, 10 | *Dendrocygna autumnalis* |  |  | M | M |  | X | R |
| 2 | *Anser caerulescens* |  |  |  |  |  |  | MI |
| 2 | *Anser albifrons* |  |  |  |  |  |  | MI |
| 3 | *Branta bernicla* |  |  | X | X | X | X | MI |
| 2, 3 | *Branta canadensis* |  |  | X | X | X | X | MI |
| 2 | *Cygnus columbianus* | X |  |  |  |  |  | MI |
| 10 | *Cairina moschata* | X | X | M | M |  |  | R |
| 1, 2, 7, 9, 10 | *Spatula discors* |  |  | M | M |  |  | MI |
| 1, 2, 3, 4 | *Spatula cyanoptera* |  | X |  |  |  |  | MI, R |
| 2, 3, 4 | *Spatula clypeata* |  |  |  |  |  |  | MI |
| 3, 4 | *Mareca strepera* |  |  | X | X |  | X | MI |
| 2, 4, 7, 9 | *Mareca americana* |  |  |  |  |  |  | MI |
| 1, 2, 3, 7, 9 | *Anas platyrhynchos* |  |  | M | M |  | X | MI, R |
| 2, 4 | *Anas diazi* | X | X |  |  |  |  | R, MI |
| 2, 4, 9 | *Anas acuta* |  | X | X |  |  |  | MI |
| 2, 4, 7 | *Anas crecca* |  |  |  |  |  |  | MI |
| 2, 3, 4 | *Aythya valisineria* |  |  | X | X |  | X | MI |
| 3, 4 | *Aythya americana* |  |  | X | X |  | X | MI, R |
| 2, 3, 4 | *Aythya collaris* |  |  |  |  |  |  | MI |
| 2, 3, 4, 7 | *Aythya affinis* |  |  |  |  |  |  | MI |
| 3 | *Histrionicus histrionicus* |  | X | X | X |  | X | A |
| 3 | *Melanitta perspicillata* |  | X | X | X |  | X | MI |
| 3 | *Clangula hyemalis* |  | X |  |  |  |  | A |
| 2, 3, 4 | *Bucephala albeola* |  |  | X | X |  | X | MI |
| 3 | *Bucephala clangula* |  |  |  |  |  |  | MI |
| 2 | *Mergus merganser* |  |  |  |  |  |  | MI |
| 3 | *Mergus serrator* |  |  | X | X |  | X | MI |
| 1, 2, 4, 7, 9 | *Oxyura jamaicensis* |  |  |  |  |  |  | MI, R |
|  | **Galliformes** |  |  |  |  |  |  |  |
|  | **Cracidae** |  |  |  |  |  |  |  |
| 4, 5, 6, 8, 9, 10 | *Ortalis vetula* |  |  | M | M | M |  | R |
| 4, 7 | *Ortalis poliocephala* | X | X | M | M |  |  | R |
| 6, 8, 9, 10 | *Penelope purpurascens* | X | X | M | M | M |  | R |
| 9 | *Penelopina nigra* | X | X | X | X |  |  | R |
| 6, 8, 9, 10 | *Crax rubra* | X | X | M | M | M |  | R |
|  | **Odontophoridae** |  |  |  |  |  |  |  |
| 1 | *Oreortyx pictus* |  | X | X | X |  |  | R |
| 4, 6, 7, 8 | *Dendrortyx macroura* | X | X | M | M | M |  | R |
| 5, 8 | *Dendrortyx barbatus* | X | X | M | M | M |  | R |
| 2, 3, 9 | *Colinus virginianus* |  | X | M | M | X |  | R |
| 10 | *Colinus nigrogularis* | X | X | X | X |  |  | R |
| 2 | *Callipepla squamata* |  | X | X | X |  |  | R |
| 1 | *Callipepla californica* |  |  | X | X |  |  | R |
| 2, 3 | *Callipepla gambelii* |  |  | M | M | X | X | R |
| 2, 4, 7, 8 | *Cyrtonyx montezumae* | X | X | M | M | M |  | R |
| 6, 9, 10 | *Dactylortyx thoracicus* | X | X | M | M | X |  | R |
| 5, 6 | *Odontophorus guttatus* | X | X | M | M | M |  | R |
|  | **Phasianidae** |  |  |  |  |  |  |  |
| 2, 3, 4, 7, 9, 10 | *Meleagris gallopavo* |  |  | M | M |  |  | R |
| 10 | *Meleagris ocellata* | X | X | X | X |  |  | R |
| 3 | *Pavo cristatus* |  |  |  |  |  |  |  |
| 1, 2, 3, 4, 7, 9, 10 | *Gallus gallus* |  |  |  |  |  |  |  |
|  | **Phoenicopteriformes** |  |  |  |  |  |  |  |
|  | **Podicipedidae** |  |  |  |  |  |  |  |
| 9, 10 | *Tachybaptus dominicus* | X |  | X |  | X |  | R |
| 1, 2, 4, 9 | *Podilymbus podiceps* |  |  | X | X |  |  | R, MI |
| 2, 3, 4 | *Podiceps nigricollis* |  |  | X | X | X |  | MI, R |
| 2, 3, 4 | *Aechmophorus occidentalis* |  | X | X |  |  |  | R, MI |
| 4 | *Aechmophorus clarkii* |  | X |  |  |  |  | R, MI |
|  | **Columbiformes** |  |  |  |  |  |  |  |
|  | **Columbidae** |  |  |  |  |  |  |  |
| 1, 2, 3, 4, 5, 7, 9, 10 | *Columba livia* |  |  | M | M | M |  | R |
| 10 | *Patagioenas speciosa* | X |  | X |  | X |  | R |
| 2, 3, 5, 6, 8, 9, 10 | *Patagioenas flavirostris* |  | X | M | M | X |  | R |
| 2, 4, 7, 9 | *Patagioenas fasciata* |  | X | M | M |  |  | R, MI |
| 6, 10 | *Patagioenas nigrirostris* | X | X | M | X | X |  | R |
| 1, 3 | *Streptopelia decaocto* |  |  |  |  |  |  | R |
| 2, 3, 4, 5, 7, 8, 9 | *Columbina inca* |  |  | M | M | M |  | R |
| 1, 2, 3, 4, 7, 9, 10 | *Columbina passerina* |  |  | M | M | M |  | R |
| 9 | *Columbina minuta* |  |  |  |  |  |  | R |
| 9, 10 | *Columbina talpacoti* |  |  | X | X |  |  | R |
| 5, 6, 10 | *Claravis pretiosa* |  |  | M | M | X |  | R |
| 10 | *Geotrygon montana* |  | X | X | X |  |  | R |
| 4, 5, 7, 8, 9, 10 | *Leptotila verreauxi* |  |  | M | M | M |  | R |
| 10 | *Leptotila jamaicensis* | X | X | X | X | X |  | R |
| 10 | *Leptotila plumbeiceps* |  | X | X | X | X |  | R |
| 6, 9, 10 | *Zentrygon albifacies* | X | X | X | X |  |  | R |
| 1, 2, 3, 4, 6, 7, 8, 9, 10 | *Zenaida asiatica* |  |  | M | M | M | M | R, MI |
| 1, 2, 3, 4, 5, 7, 9, 10 | *Zenaida macroura* |  |  | M | M | M | X | R, MI |
|  | **Cuculidae** |  |  |  |  |  |  |  |
| 2, 5, 9, 10 | *Crotophaga sulcirostris* |  |  | M |  | X | M | R |
| 10 | *Tapera naevia* |  |  |  |  |  |  | R |
| 10 | *Dromococcyx phasianellus* |  | X | X |  |  |  | R |
| 4, 6, 7, 8, 9, 10 | *Geococcyx velox* |  |  | M | M | M |  | R |
| 1, 2, 3 | *Geococcyx californianus* |  |  | M |  | M |  | R |
| 4, 5, 6, 8, 9, 10 | *Piaya cayana* |  |  | M | M | X |  | R |
| 2, 9 | *Coccyzus americanus* |  | X | M | X | M |  | MV, T |
| 10 | *Coccyzus minor* |  | X |  |  |  |  | R |
|  | **Caprimulgiformes** |  |  |  |  |  |  |  |
|  | **Caprimulgidae** |  |  |  |  |  |  |  |
| 1, 2, 3, 4, 6, 7, 8, 9, 10 | *Chordeiles acutipennis* |  |  | M | M | M |  | MV, MI, R |
| 4, 9, 10 | *Chordeiles minor* |  |  |  |  | X |  | MV, T |
| 9, 10 | *Nyctidromus albicollis* |  |  | M | X | M |  | R |
| 1, 2, 3 | *Phalaenoptilus nuttallii* |  |  | X |  | X |  | R |
| 10 | *Nyctiphrynus yucatanicus* | X | X | X |  |  |  | R |
| 10 | *Antrostomus salvini* | X | X |  |  |  |  | R |
| 8 | *Antrostomus vociferus* |  | X | M | M | M |  | MI |
| 4, 6, 7, 9 | *Antrostomus arizonae* |  | X | M | X | X |  | R, MV |
|  | **Nyctibiiformes** |  |  |  |  |  |  |  |
|  | **Nyctibiidae** |  |  |  |  |  |  |  |
| 9, 10 | *Nyctibius jamaicensis* |  | X | X | X |  |  | R |
|  | **Apodiformes** |  |  |  |  |  |  |  |
|  | **Apodidae** |  |  |  |  |  |  |  |
| 3, 4, 6, 7, 9 | *Cypseloides niger* |  | X | M | X | M |  | MV,T |
| 4, 6, 7, 8, 9 | *Streptoprocne rutila* |  |  | M | X | M |  | R |
| 5, 6, 8, 9 | *Streptoprocne zonaris* |  |  | M | M | M |  | R |
| 4 | *Streptoprocne semicollaris* | X | X |  |  |  |  | R |
| 10 | *Chaetura pelagica* |  | X | X | X |  |  | T |
| 2, 4, 5, 9, 10 | *Chaetura vauxi* |  | X | M | X | M |  | R, T |
| 1, 2, 4, 7, 9 | *Aeronautes saxatalis* |  |  | M |  | M |  | R |
| 6 | *Panyptila cayennensis* | X | X | X | X | X |  | R |
| 4, 9 | *Panyptila sanctihieronymi* | X | X |  |  |  |  | R |
|  | **Trochilidae** |  |  |  |  |  |  |  |
| 4, 6, 8 | *Phaethornis longirostris* |  | X | M | M | M |  | R |
| 5, 6, 9, 10 | *Phaethornis striigularis* | X | X | M | M | M |  | R |
| 4, 7, 9 | *Colibri thalassinus* |  | X | M | M | M |  | R |
| 5 | *Anthracothorax prevostii* |  |  | X | X | X |  | R, MV, MI |
| 4, 5, 7, 8, 9 | *Eugenes fulgens* |  | X | M | M | M |  | R |
| 9 | *Heliomaster constantii* |  | X |  |  |  |  | R |
| 4, 5, 6, 7, 8, 9 | *Lampornis amethystinus* |  | X | M | M | M |  | R |
| 4, 7, 8 | *Lampornis clemenciae* | X | X | M | M | M |  | R |
| 6, 7, 8, 9 | *Lamprolaima rhami* | X | X | M | M | M |  | R |
| 9 | *Doricha enicura* | X | X | X | X | X |  | R |
| 4, 8 | *Tilmatura dupontii* | X | X | X | X | X |  | R |
| 4 | *Calothorax lucifer* | X | X |  |  |  |  | MV, MI, R |
| 4, 7, 9 | *Calothorax pulcher* | X | X | M | M | M |  | R |
| 4, 5, 7, 8, 9, 10 | *Archilochus colubris* |  |  | M | M | M |  | MI, T |
| 2, 3, 4 | *Archilochus alexandri* | X |  | X | X | X |  | MV, MI |
| 1, 2 | *Calypte anna* |  |  | X | X | X |  | MI, R |
| 1, 2, 3 | *Calypte costae* |  |  | M | M | M |  | R, MI |
| 3, 4 | *Selasphorus calliope* | X | X |  |  |  |  | MI, MV |
| 2, 3, 4, 7, 8, 9 | *Selasphorus rufus* |  | X | M | M | M |  | MI |
| 3, 4 | *Selasphorus sasin* | X | X |  |  |  |  | MI, MV |
| 4, 6, 7, 9 | *Selasphorus platycercus* | X | X | M | M | M |  | R, MI, MV |
| 4, 5, 6, 7, 8 | *Selasphorus heloisa* | X | X | M | M | M |  | R |
| 9 | *Selasphorus ellioti* | X | X | X | X | X |  | R |
| 7, 8 | *Phaeoptila sordida* | X | X | M | M | M |  | R |
| 2,3,4,7, | *Cynanthus latirostris* | X |  | M | M | M |  | R |
| 5, 9, 10 | *Cynanthus canivetii* |  |  | M | M | M |  | R |
| 2, 4, 5, 6, 7, 8, 9 | *Basilinna leucotis* |  |  | M | M | M |  | R |
| 5 | *Basilinna xantusii* | X | X |  |  |  |  | R |
| 5, 6, 8, 10 | *Pampa curvipennis* |  | X | M | M | M |  | R |
| 6 | *Abeillia abeillei* | X | X | X | X | X |  | R |
| 6, 8 | *Campylopterus hemileucurus* |  | X | M | M | M |  | R |
| 6, 8 | *Eupherusa eximia* |  | X | M | M | M |  | R |
| 2, 4 | *Ramosomyia violiceps* | X |  |  |  |  |  | R |
| 7 | *Ramosomyia viridifrons* | X | X | X | X | X |  | R |
| 5, 6, 8, 9 | *Saucerottia cyanocephala* |  |  | M | M | M |  | R |
| 2, 4, 6, 7, 8, 9 | *Saucerottia beryllina* |  |  | M | M | M |  | R |
| 10 | *Amazilia rutila* |  |  | X | X |  |  | R |
| 5, 10 | *Amazilia yucatanensis* | X |  | M | M | X |  | R |
| 9, 10 | *Amazilia tzacatl* |  |  | M | M | X |  | R |
| 5, 6, 10 | *Chlorestes candida* |  | X | M | M | M |  | R, MI |
|  | **Gruiformes** |  |  |  |  |  |  |  |
|  | **Rallidae** |  |  |  |  |  |  |  |
| 5, 10 | *Aramides albiventris* |  | X | M | X |  |  | R |
| 2, 3 | *Rallus obsoletus* |  | X | X |  |  |  | R |
| 4 | *Rallus elegans* | X | X |  |  |  |  | R |
| 1, 2, 3, 4, 9 | *Rallus limicola* | X |  | X |  |  |  | MI, R |
| 1, 2, 4, 9, 10 | *Porzana carolina* |  |  |  |  |  |  | MI, R |
| 1, 2, 4, 10 | *Gallinula galeata* |  |  |  |  |  |  | R, MI |
| 1, 2, 3, 4, 9, 10 | *Fulica americana* |  |  | M |  | X |  | R, MI |
| 10 | *Porphyrio martinicus* |  |  |  |  |  |  | R, MI |
| 2 | *Coturnicops noveboracensis* |  | X |  |  |  |  | R |
| 10 | *Laterallus ruber* |  |  |  |  |  |  | R |
|  | **Aramidae** |  |  |  |  |  |  |  |
| 10 | *Aramus guarauna* | X |  | X | X |  |  | R |
|  | **Gruidae** |  |  |  |  |  |  |  |
| 2 | *Antigone canadensis* | X |  | X |  | X |  | MI |
|  | **Charadriiformes** |  |  |  |  |  |  |  |
|  | **Recurvirostridae** |  |  |  |  |  |  |  |
| 1, 2, 3, 4, 10 | *Himantopus mexicanus* |  |  | X |  |  |  | R, MI |
| 2, 3, 4 | *Recurvirostra americana* |  |  | X |  |  |  | MI, R |
|  | **Haematopodidae** |  |  |  |  |  |  |  |
| 1, 2, 3 | *Haematopus palliatus* |  | X | M |  |  |  | R, MI |
|  | **Charadriidae** |  |  |  |  |  |  |  |
| 3 | *Pluvialis squatarola* |  |  | X |  |  |  | MI |
| 1, 2, 3, 4, 9, 10 | *Charadrius vociferus* |  |  | M | M | M |  | R, MI |
| 1, 3, 9 | *Charadrius semipalmatus* |  | X | M |  | X |  | MI |
| 1, 3 | *Charadrius wilsonia* |  | X | X |  |  |  | R, MI |
| 2, 3 | *Charadrius montanus* | X | X |  |  |  |  | MI |
| 1, 2, 3, 4 | *Charadrius nivosus* | X | X | X |  |  |  | MI, MV, R |
|  | **Jacanidae** |  |  |  |  |  |  |  |
| 10 | *Jacana spinosa* |  |  |  |  |  |  | R |
|  | **Scolopacidae** |  |  |  |  |  |  |  |
| 7 | *Bartramia longicauda* |  |  |  |  |  |  | T |
| 3 | *Numenius phaeopus* |  | X | X |  | X |  | MI |
| 2, 3, 4 | *Numenius americanus* |  | X | X |  |  |  | MI |
| 3 | *Limosa fedoa* | X | X |  |  |  |  | MI |
| 3 | *Arenaria interpres* |  | X | X | X |  |  | MI |
| 3 | *Arenaria melanocephala* |  | X | X | X |  |  | MI |
| 3 | *Calidris canutus* |  | X |  |  |  |  | MI |
| 3 | *Calidris virgata* |  | X |  |  |  |  | MI |
| 4 | *Calidris himantopus* |  | X |  |  |  |  | MI, T |
| 3 | *Calidris alba* |  |  | X | X |  |  | MI |
| 3 | *Calidris alpina* |  |  |  |  |  |  | MI |
| 2, 3, 4, 9 | *Calidris minutilla* |  |  | X |  | X |  | MI |
| 2, 9 | *Calidris melanotos* |  | X |  |  |  |  | T, MI |
| 2 | *Calidris pusilla* |  | X |  |  |  |  | T, MI |
| 2, 3, 4 | *Calidris mauri* | X | X |  |  |  |  | MI, T |
| 2, 3, 4 | *Limnodromus scolopaceus* |  | X |  |  |  |  | MI |
| 2, 4, 10 | *Gallinago delicata* |  |  |  |  |  |  | MI |
| 2, 3, 4, 9 | *Actitis macularius* |  |  | X |  |  |  | MI |
| 2, 3, 4, 9, 10 | *Tringa solitaria* |  |  |  |  |  |  | MI |
| 3, 4 | *Tringa flavipes* |  | X |  |  |  |  | MI |
| 3 | *Tringa semipalmata* |  | X |  |  |  |  | MI |
| 2, 3, 4 | *Tringa melanoleuca* |  |  | X | X |  |  | MI |
| 2, 3 | *Phalaropus tricolor* |  |  |  |  |  |  | T, MI |
| 2, 3, 7 | *Phalaropus lobatus* |  |  |  |  | X |  | MI |
| 3 | *Phalaropus fulicarius* |  | X | X |  |  |  | MI |
|  | **Stercorariidae** |  |  |  |  |  |  |  |
| 3 | *Stercorarius pomarinus* |  |  | X |  |  |  | T, MI, O |
| 3 | *Stercorarius parasiticus* |  |  |  |  |  |  | T, O |
|  | **Alcidae** |  |  |  |  |  |  |  |
| 3 | *Synthliboramphus craveri* | X | X |  |  |  |  | R, O |
|  | **Laridae** |  |  |  |  |  |  |  |
| 2, 3 | *Chroicocephalus philadelphia* |  |  |  |  |  |  | MI |
| 3, 4, 9, 10 | *Leucophaeus atricilla* |  |  |  |  |  |  | MI, R |
| 4 | *Leucophaeus pipixcan* |  | X |  |  |  |  | T |
| 1, 3 | *Larus heermanni* | X | X | X | X |  | X | R |
| 2, 3, 4 | *Larus delawarensis* |  |  | M | M | X | X | MI |
| 3 | *Larus occidentalis* |  | X |  |  |  |  | R, MI |
| 1, 2, 3 | *Larus livens* | X | X | X | X | X | X | R |
| 2, 3 | *Larus californicus* |  | X | X | X |  | X | MI |
| 2, 3 | *Larus argentatus* |  |  | X |  | X | X | MI |
| 1 | *Sternula antillarum* | X | X |  |  |  |  | R, MV |
| 1 | *Gelochelidon nilotica* |  | X |  |  |  |  | R, MI |
| 3, 4 | *Hydroprogne caspia* |  | X | X | X |  |  | MI, R |
| 2, 3 | *Chlidonias niger* |  | X |  |  |  |  | T, MI |
| 3 | *Sterna hirundo* |  |  |  |  |  |  | MI, T |
| 3 | *Sterna forsteri* |  | X |  |  |  |  | MI, R |
| 3 | *Thalasseus maximus* |  |  |  |  |  |  | MI, R |
| 3 | *Thalasseus sandvicensis* |  |  |  |  |  |  | MI, R |
| 3 | *Thalasseus elegans* | X | X |  |  |  |  | MV, MI |
| 3 | *Rynchops niger* |  | X |  |  |  |  | MI, R |
|  | **Phaethontiformes** |  |  |  |  |  |  |  |
|  | **Phaethontidae** |  |  |  |  |  |  |  |
| 3 | *Phaethon aethereus* | X | X |  |  |  |  | R, O |
|  | **Gaviiformes** |  |  |  |  |  |  |  |
|  | **Gaviidae** |  |  |  |  |  |  |  |
| 3 | *Gavia stellata* |  |  | X | X | X | X | MI |
| 3 | *Gavia pacifica* |  |  |  |  |  |  | MI |
| 3 | *Gavia immer* |  |  | X | X | X | X | MI |
| 3 | *Gavia adamsii* |  |  |  |  |  |  | A |
|  | **Procellariiformes** |  |  |  |  |  |  |  |
|  | **Hydrobatidae** |  |  |  |  |  |  |  |
| 3 | *Hydrobates melania* | X | X |  |  |  |  | R, O |
| 3 | *Hydrobates microsoma* | X | X | X |  | X |  | R, MI, O |
|  | **Procellariidae** |  |  |  |  |  |  |  |
| 3 | *Fulmarus glacialis* |  |  |  |  |  |  | MI, O |
| 3 | *Ardenna grisea* |  | X |  |  |  |  | T, O |
| 3 | *Ardenna creatopus* | X | X |  |  |  |  | T, O |
| 3 | *Puffinus opisthomelas* | X | X |  |  |  |  | R, O |
|  | **Ciconiiformes** |  |  |  |  |  |  |  |
|  | **Ciconiidae** |  |  |  |  |  |  |  |
| 2, 3, 10 | *Mycteria americana* | X | X | M | M | X |  | MI, R |
|  | **Suliformes** |  |  |  |  |  |  |  |
|  | **Fregatidae** |  |  |  |  |  |  |  |
| 3, 10 | *Fregata magnificens* |  | X | X |  |  |  | R |
|  | **Sulidae** |  |  |  |  |  |  |  |
| 3 | *Sula nebouxii* | X | X | X | X |  |  | R |
| 3 | *Sula leucogaster* |  | X | X | X | X |  | R |
|  | **Anhingidae** |  |  |  |  |  |  |  |
| 10 | *Anhinga anhinga* |  |  |  |  |  |  | R |
|  | **Phalacrocoracidae** |  |  |  |  |  |  |  |
| 3 | *Phalacrocorax penicillatus* |  | X | X |  |  |  | R |
| 1, 2, 3 | *Phalacrocorax auritus* |  |  | X | X | X |  | MI, R |
| 10 | *Phalacrocorax brasilianus* |  |  |  |  |  |  | R |
|  | **Pelecaniformes** |  |  |  |  |  |  |  |
|  | **Pelecanidae** |  |  |  |  |  |  |  |
| 2, 3 | *Pelecanus erythrorhynchos* |  |  | X | X | X | X | MI |
| 1, 2, 3 | *Pelecanus occidentalis* |  |  | M | X | X | X | R, MI |
|  | **Ardeidae** |  |  |  |  |  |  |  |
| 10 | *Botaurus pinnatus* | X |  | X |  |  |  | R |
| 2, 3, 4 | *Botaurus lentiginosus* | X | X | X |  | X |  | MI, R |
| 2, 3 | *Ixobrychus exilis* | X |  |  |  |  |  | MI, R |
| 10 | *Tigrisoma mexicanum* | X | X | X |  |  |  | R |
| 1, 2, 3, 4, 9, 10 | *Ardea herodias* |  |  | M | M | M |  | MI, R |
| 2, 3, 4, 8, 9, 10 | *Ardea alba* |  |  | M | M |  |  | MI, R |
| 2, 3, 4, 9, 10 | *Egretta thula* |  |  | M | X | M |  | MI, R |
| 3, 4, 9, 10 | *Egretta caerulea* |  | X | M | X |  |  | MI, R |
| 3, 4, 10 | *Egretta tricolor* |  | X | M | X |  |  | MI, R |
| 3 | *Egretta rufescens* | X | X | X | X | X | X | MI, R |
| 2, 4, 7, 10 | *Bubulcus ibis* |  |  |  |  |  |  | R, MI |
| 1, 2, 3, 4, 9, 10 | *Butorides virescens* |  | X | M |  |  |  | R, MI |
| 2, 3, 4, 9, 10 | *Nycticorax nycticorax* |  |  | X |  |  |  | R, MI |
| 3, 4, 10 | *Nyctanassa violacea* |  | X | M |  |  | X | R, MI |
|  | **Threskiornithidae** |  |  |  |  |  |  |  |
| 3, 10 | *Eudocimus albus* |  | X | X |  |  |  | R, MI |
| 2, 3, 4 | *Plegadis chihi* |  |  | X |  |  |  | MI, R |
| 2, 3, 10 | *Platalea ajaja* |  |  | X |  |  |  | MI, R |
|  | **Cathartiformes** |  |  |  |  |  |  |  |
|  | **Cathartidae** |  |  |  |  |  |  |  |
| 1 | *Gymnogyps californianus* | X | X | X | X | X | X | R |
| 6, 7, 9, 10 | *Sarcoramphus papa* | X | X | M | X | X | M | R |
| 2, 3, 4, 5, 6, 7, 8, 9, 10 | *Coragyps atratus* |  |  | M | M | M | M | R |
| 1, 2, 3, 4, 5, 6, 7, 8, 9, 10 | *Cathartes aura* |  |  | M | M | M | M | R |
|  | **Accipitriformes** |  |  |  |  |  |  |  |
|  | **Pandionidae** |  |  |  |  |  |  |  |
| 1, 2, 3, 10 | *Pandion haliaetus* |  |  | M | X | X | X | MI, R |
|  | **Accipitridae** |  |  |  |  |  |  |  |
| 1, 3, 9, 10 | *Elanus leucurus* |  |  | M |  |  | M | R |
| 4, 7, 10 | *Chondrohierax uncinatus* | X | X | X |  |  |  | MI, R |
| 9, 10 | *Elanoides forficatus* | X | X | X |  |  | X | T, MV |
| 9, 10 | *Harpia harpyja* | X | X | M |  |  | X | R |
| 1, 2, 3, 4 | *Aquila chrysaetos* | X |  | M | M | M | M | MI, R |
| 6, 10 | *Spizaetus tyrannus* | X | X | M | X | X | X | R |
| 10 | *Spizaetus ornatus* | X | X | X |  |  |  | R |
| 1, 2, 3, 4, 7, 10 | *Circus hudsonius* |  |  | M | X | M | M | MI, R |
| 2, 3, 4, 5, 6, 7, 9 | *Accipiter striatus* | X |  | M | M | M | M | MI, R |
| 1, 2, 3, 4, 5, 7, 8 | *Accipiter cooperii* | X |  | M | M | M | M | MI, R |
| 10 | *Ictinia plumbea* | X |  | X |  |  |  | MV |
| 10 | *Rostrhamus sociabilis* | X |  |  |  |  |  | R |
| 4, 8, 10 | *Buteogallus anthracinus* | X |  | M | X | X | X | R, MV |
| 10 | *Buteogallus urubitinga* | X |  | X |  |  |  | R |
| 4, 6 | *Buteogallus solitarius* | X | X | X | X | X | X | R |
| 5, 6, 9, 10 | *Rupornis magnirostris* |  |  | M | M | X | M | R |
| 1, 2, 3 | *Parabuteo unicinctus* | X |  | X | X |  |  | R |
| 8, 9 | *Geranoaetus albicaudatus* | X |  | X | X | X | X | R |
| 6 | *Pseudastur albicollis* | X | X | X | X | X | X | R |
| 4, 5, 10 | *Buteo plagiatus* |  |  | M | X |  | X | R |
| 1 | *Buteo lineatus* | X |  |  |  |  |  | MI, R |
| 5 | *Buteo platypterus* | X |  | X | X |  | X | T, MI |
| 4, 5, 7, 9, 10 | *Buteo brachyurus* |  |  | M | M | X | M | R |
| 2, 3 | *Buteo swainsoni* | X |  |  |  |  |  | T, MV |
| 1, 2, 3, 6, 7, 8, 10 | *Buteo albonotatus* | X |  | M | M | M | M | MI, MV, R |
| 1, 2, 3, 4, 5, 6, 7, 8, 9 | *Buteo jamaicensis* |  |  | M | M | M | M | R, MI |
| 2 | *Buteo regalis* | X |  |  |  |  |  | MI |
|  | **Strigiformes** |  |  |  |  |  |  |  |
|  | **Tytonidae** |  |  |  |  |  |  |  |
| 1, 2, 3, 4, 7, 9, 10 | *Tyto alba* |  |  | M | M | M | M | R |
|  | **Strigidae** |  |  |  |  |  |  |  |
| 2, 4, 8 | *Psiloscops flammeolus* | X | X | M | M | M | M | R, MV, MI |
| 4, 7, 9 | *Megascops trichopsis* |  | X | M | M | M | X | R |
| 9 | *Megascops barbarus* | X | X |  |  |  |  | R |
| 1, 2, 3, 4 | *Megascops kennicottii* |  | X | M |  | M | X | R |
| 9, 10 | *Megascops guatemalae* |  | X | X |  |  |  | R |
| 1, 2, 3, 4, 7, 9, 10 | *Bubo virginianus* |  |  | M | M | M | M | R |
| 4, 7, 8, 9 | *Glaucidium gnoma* |  |  | M | M | M | M | R |
| 2, 3, 5, 9, 10 | *Glaucidium brasilianum* |  |  | M | X | M | X | R |
| 2, 3, 7 | *Micrathene whitneyi* | X | X | X | X | M | M | MI, R, MV |
| 1, 2, 3, 4 | *Athene cunicularia* |  |  | M |  | X | X | R, MI |
| 4, 6, 7, 8, 9, 10 | *Ciccaba virgata* |  |  | M | M | M | M | R |
| 1 | *Strix occidentalis* | X | X |  |  |  |  | R |
| 7, 9 | *Strix fulvescens* | X | X | X |  | X |  | R |
| 1, 2, 3, 4 | *Asio otus* |  | X | M |  | M | X | MI, R |
| 8 | *Asio stygius* | X | X | X | X | X | X | R |
| 2, 3, 4 | *Asio flammeus* | X | X |  |  |  |  | MI |
| 4 | *Aegolius acadicus* |  |  |  |  |  |  | R |
| 9 | *Aegolius ridgwayi* | X | X | X |  | X |  | R |
|  | **Trogoniformes** |  |  |  |  |  |  |  |
|  | **Trogonidae** |  |  |  |  |  |  |  |
| 6 | *Trogon massena* | X | X | X | X |  | X | R |
| 6, 10 | *Trogon melanocephalus* |  | X | M | X | X | X | R |
| 5, 6, 8, 9, 10 | *Trogon caligatus* |  |  | M | M | M | X | R |
| 2, 4, 7 | *Trogon elegans* |  | X | M | X | X | M | R |
| 4, 5, 6, 7, 8, 9 | *Trogon mexicanus* |  | X | M | M | M | M | R |
| 5, 6, 8, 10 | *Trogon collaris* | X |  | M | M | M | M | R |
| 2 | *Euptilotis neoxenus* | X | X |  |  |  |  | R |
|  | **Momotidae** |  |  |  |  |  |  |  |
| 9 | *Aspatha gularis* | X | X | X |  |  |  | R |
| 5, 8, 9 | *Momotus mexicanus* | X | X | M | X | X |  | R |
| 5 | *Momotus coeruliceps* | X | X | X | X | X |  | R |
| 5, 6, 9, 10 | *Momotus lessonii* |  |  | M | M | X |  | R |
| 10 | *Eumomota superciliosa* |  | X | X |  |  |  | R |
|  | **Alcedinidae** |  |  |  |  |  |  |  |
| 9, 10 | *Megaceryle torquata* |  |  |  |  |  |  | R |
| 2, 3, 4 | *Megaceryle alcyon* |  |  | M |  | X |  | MI |
| 2, 3, 4, 8, 10 | *Chloroceryle americana* |  |  | X | X | X |  | R |
|  | **Piciformes** |  |  |  |  |  |  |  |
|  | **Bucconidae** |  |  |  |  |  |  |  |
| 10 | *Notharchus hyperrhynchus* | X |  | X |  |  |  | R |
|  | **Ramphastidae** |  |  |  |  |  |  |  |
| 5, 6, 7, 8, 9 | *Aulacorhynchus prasinus* | X | X | M | M | X |  | R |
| 5, 6, 9, 10 | *Pteroglossus torquatus* | X | X | M | M |  |  | R |
| 5, 6, 8, 9, 10 | *Ramphastos sulfuratus* | X | X | M | M | X |  | R |
|  | **Picidae** |  |  |  |  |  |  |  |
| 2, 3 | *Melanerpes lewis* |  | X |  |  |  |  | MI |
| 1, 2, 4, 6, 7, 8, 9 | *Melanerpes formicivorus* |  |  | M | M | M |  | R |
| 7, 8 | *Melanerpes hypopolius* | X | X | M | X | M |  | R |
| 10 | *Melanerpes pygmaeus* | X | X | X |  |  |  | R |
| 1, 2, 3 | *Melanerpes uropygialis* |  |  | M | X | M | X | R |
| 5, 6, 8, 9, 10 | *Melanerpes aurifrons* |  |  | M | M | M |  | R |
| 2 | *Sphyrapicus thyroideus* |  | X |  |  |  |  | MI, R |
| 3, 4, 7 | *Sphyrapicus varius* |  |  | X |  | X |  | MI |
| 2, 3 | *Sphyrapicus nuchalis* |  |  | X | X | X | X | MI |
| 2 | *Sphyrapicus ruber* |  |  |  |  |  |  | MI |
| 1 | *Dryobates nuttallii* |  |  |  |  |  |  | R |
| 1, 2, 3, 4, 5, 6, 7, 8, 9, 10 | *Dryobates scalaris* |  |  | M | M | M | X | R |
| 1, 4, 7, 8, 9 | *Dryobates villosus* |  |  | M | M | M |  | R |
| 5, 6, 10 | *Dryobates fumigatus* |  | X | M | M |  |  | R |
| 4 | *Dryobates stricklandi* | X | X | X | X |  |  | R |
| 5, 6, 9, 10 | *Colaptes rubiginosus* |  |  | M | M | X |  | R |
| 1, 2, 3, 4, 7, 9 | *Colaptes auratus* | X | X | M | M | M | X | R, MI |
| 2, 3 | *Colaptes chrysoides* |  | X | X | X | X | X | R |
| 5, 10 | *Celeus castaneus* | X | X | M | X |  |  | R |
| 5, 6, 7, 9, 10 | *Dryocopus lineatus* |  |  | M | M | M |  | R |
| 6, 8, 9, 10 | *Campephilus guatemalensis* | X | X | M | M | M |  | R |
|  | **Falconiformes** |  |  |  |  |  |  |  |
|  | **Falconidae** |  |  |  |  |  |  |  |
| 9, 10 | *Herpetotheres cachinnans* |  |  | X |  |  |  | R |
| 6, 8, 10 | *Micrastur ruficollis* | X | X | M | M | M | M | R |
| 5, 6, 8, 10 | *Micrastur semitorquatus* | X |  | M | M | M | M | R |
| 2, 3, 4, 7 | *Caracara plancus* |  |  | M |  | X |  | R |
| 1, 2, 3, 4, 5, 6, 7, 8, 9, 10 | *Falco sparverius* |  |  | M | M | M | M | R, MI |
| 2, 4, 8 | *Falco columbarius* |  |  | X | X | X | X | MI |
| 10 | *Falco rufigularis* |  |  | X |  |  |  | R |
| 1, 2, 3, 4, 7 | *Falco peregrinus* | X |  | M | X | X |  | R, MI |
| 2, 3 | *Falco mexicanus* | X |  | M | X | X |  | R, MI |
|  | **Psittaciformes** |  |  |  |  |  |  |  |
|  | **Psittacidae** |  |  |  |  |  |  |  |
| 6, 10 | *Eupsittula nana* | X | X | M | M | X |  | R |
| 7, 9 | *Eupsittula canicularis* | X | X | M | M |  |  | R |
| 9, 10 | *Ara macao* | X | X | X | X | X |  | R |
| 7, 8, 9 | *Ara militaris* | X | X | M | M | X |  | R |
| 6, 9 | *Psittacara holochlorus* | X | X | X | X | X |  | R |
| 9 | *Psittacara strenuus* | X | X |  |  |  |  | R |
| 2 | *Rhynchopsitta pachyrhyncha* | X | X |  |  |  |  | MV, MI |
| 8, 9 | *Bolborhynchus lineola* | X | X | X | X | X |  | R |
| 9 | *Brotogeris jugularis* | X | X |  |  |  |  | R |
| 6 | *Pyrilia haematotis* | X | X | X | X | X |  | R |
| 5, 6, 8, 9, 10 | *Pionus senilis* | X | X | M | M | M |  | R |
| 2, 5, 9, 10 | *Amazona albifrons* | X | X | M | X | M |  | R |
| 10 | *Amazona xantholora* | X | X | X |  |  |  | R |
| 7 | *Amazona finschi* | X | X |  |  |  |  | R |
| 5, 8, 10 | *Amazona autumnalis* | X | X | M | M | M |  | R |
| 6, 9 | *Amazona oratrix* | X | X | X | X | X |  | R |
|  | **Passeriformes** |  |  |  |  |  |  |  |
|  | **Pipridae** |  |  |  |  |  |  |  |
| 6 | *Ceratopipra mentalis* |  | X | X | X |  |  | R |
|  | **Cotingidae** |  |  |  |  |  |  |  |
| 6 | *Cotinga amabilis* | X | X | X | X |  |  | R |
|  | **Tityridae** |  |  |  |  |  |  |  |
| 5, 6, 9, 10 | *Tityra semifasciata* |  |  | X | X |  |  | R |
| 6, 10 | *Tityra inquisitor* |  |  | X | X |  |  | R |
| 2, 6, 7, 10 | *Pachyramphus major* |  | X | X | X |  |  | R |
| 2, 4, 6, 7, 8, 9, 10 | *Pachyramphus aglaiae* |  |  | M | M |  |  | R |
|  | **Onychorhynchidae** |  |  |  |  |  |  |  |
| 10 | *Onychorhynchus coronatus* | X | X | X |  |  |  | R |
| 6, 10 | *Myiobius sulphureipygius* |  | X | X | X |  |  | R |
|  | **Tyrannidae** |  |  |  |  |  |  |  |
| 6, 8, 10 | *Platyrinchus cancrominus* | X | X | M | M |  |  | R |
| 5, 6, 8 | *Mionectes oleagineus* |  |  | M | M | X |  | R |
| 10 | *Oncostoma cinereigulare* |  | X |  |  |  |  | R |
| 6, 10 | *Rhynchocyclus brevirostris* |  | X | X | X |  |  | R |
| 5, 10 | *Tolmomyias sulphurescens* |  |  |  |  |  |  | R |
| 2, 4, 7, 8 | *Camptostoma imberbe* |  |  | X | X | X |  | R |
| 4, 5, 7, 8, 10 | *Myiopagis viridicata* |  |  | X | X | X |  | R |
| 10 | *Elaenia flavogaster* |  |  |  |  |  |  | R |
| 4, 10 | *Attila spadiceus* |  |  | X |  |  | X | R |
| 10 | *Myiarchus yucatanensis* | X | X | X |  | X |  | R |
| 4, 6, 7, 8, 9, 10 | *Myiarchus tuberculifer* |  |  | M | M | M |  | R |
| 1, 2, 3, 4 | *Myiarchus cinerascens* |  |  | M | X | M |  | MI, MV, R |
| 3, 7 | *Myiarchus nuttingi* |  | X |  |  |  |  | R |
| 8 | *Myiarchus crinitus* |  |  | X | X |  |  | MI |
| 2, 3, 4, 6, 8, 10 | *Myiarchus tyrannulus* |  |  | M | M | X |  | R, MV |
| 5, 6, 8, 9, 10 | *Pitangus sulphuratus* |  |  | M | M | X |  | R |
| 5, 6, 8, 9, 10 | *Megarynchus pitangua* |  |  | M | M | X |  | R |
| 5, 6, 8, 9, 10 | *Myiozetetes similis* |  |  | M | M | X | X | R |
| 5, 6, 10 | *Myiodynastes maculatus* |  |  | M | M |  |  | MV |
| 5, 8, 9, 10 | *Myiodynastes luteiventris* |  |  | M | M |  | X | MV |
| 5, 10 | *Legatus leucophaius* |  |  |  |  |  |  | MV |
| 3, 4, 5, 6, 8, 9 | *Tyrannus melancholicus* |  |  | M | M |  | X | R |
| 10 | *Tyrannus couchii* |  |  | X | X | X |  | R |
| 1, 2, 3, 4, 6, 7, 9 | *Tyrannus vociferans* | X |  | M | X | X |  | R, MI |
| 4, 7, 8 | *Tyrannus crassirostris* | X | X | M | X | X | X | R, MV, MI |
| 1, 2, 6, 7, 8 | *Tyrannus verticalis* |  |  | M | M | M | X | MI, T, MV |
| 9, 10 | *Tyrannus tyrannus* |  |  |  |  |  |  | T |
| 7, 9 | *Tyrannus forficatus* |  |  |  |  | X |  | MI, T, MV |
| 10 | *Tyrannus savana* |  |  |  |  |  |  | R |
| 4, 9 | *Xenotriccus callizonus* | X | X | X |  |  |  | R |
| 4, 7, 8 | *Xenotriccus mexicanus* | X | X | X | X |  | X | R |
| 4, 5, 6, 7, 8, 9 | *Mitrephanes phaeocercus* |  | X | M | X |  | X | R |
| 1, 2, 4, 10 | *Contopus cooperi* |  | X |  | X | X |  | T, MI, MV |
| 4, 5, 6, 7, 8, 9 | *Contopus pertinax* |  | X | M | M | M |  | R |
| 1, 2, 4, 6, 7, 8, 9 | *Contopus sordidulus* |  | X | M | M | X | X | MV, T |
| 9, 10 | *Contopus virens* |  |  | X |  | X |  | T |
| 9, 10 | *Contopus cinereus* |  |  | X |  | X |  | R |
| 5, 10 | *Empidonax flaviventris* |  |  | X |  |  | X | MI |
| 8, 10 | *Empidonax virescens* |  |  | X | X |  | X | T |
| 2, 3, 8 | *Empidonax traillii* |  |  | X | X |  | X | MI |
| 4, 9 | *Empidonax albigularis* |  | X | X |  |  |  | MI, MV, R |
| 2, 6, 8, 10 | *Empidonax minimus* |  |  | M | M |  | X | MI |
| 2, 3, 4, 7 | *Empidonax hammondii* |  |  |  |  |  |  | MI |
| 2, 3, 4 | *Empidonax wrightii* | X |  | X |  |  |  | MI |
| 2, 3, 4, 7, 8 | *Empidonax oberholseri* | X |  | M | X |  | X | MI |
| 4, 6, 7, 8, 9 | *Empidonax affinis* | X | X | M | M |  | X | R |
| 1, 3 | *Empidonax difficilis* | X |  |  |  |  |  | MI, R |
| 2, 4, 5, 6, 7, 8 | *Empidonax occidentalis* | X |  | M | M |  | M | R, MI, MV |
| 6, 9 | *Empidonax flavescens* |  | X | X |  |  |  | R |
| 2, 4, 7, 9 | *Empidonax fulvifrons* |  | X | X | X | X |  | R, MI, MV |
| 1, 2, 3, 4, 6, 7, 8, 9 | *Sayornis nigricans* |  |  | M | M |  | X | R, MI |
| 2, 4, 8 | *Sayornis phoebe* |  |  | X | X |  | X | MI |
| 1, 2, 3, 4, 7 | *Sayornis saya* |  |  | X | X | M |  | R, MI |
| 1, 2, 3, 4, 9, 10 | *Pyrocephalus rubinus* |  |  | X |  |  |  | R, MI |
|  | **Thamnophilidae** |  |  |  |  |  |  |  |
| 6 | *Taraba major* | X |  | X | X |  |  | R |
| 5, 6, 9, 10 | *Thamnophilus doliatus* |  |  | M | M |  |  | R |
|  | **Grallariidae** |  |  |  |  |  |  |  |
| 4 | *Grallaria guatimalensis* | X | X |  |  |  |  | R |
|  | **Formicariidae** |  |  |  |  |  |  |  |
| 6, 10 | *Formicarius moniliger* |  | X | X | X |  |  | R |
|  | **Furnariidae** |  |  |  |  |  |  |  |
| 6, 8 | *Sclerurus mexicanus* | X | X | M | M |  |  | R |
| 5, 6, 8, 10 | *Sittasomus griseicapillus* |  |  | M | M |  |  | R |
| 6, 8, 10 | *Dendrocincla homochroa* |  | X | M | M |  |  | R |
| 6, 8, 10 | *Dendrocincla anabatina* | X | X | M | M |  |  | R |
| 10 | *Dendrocolaptes sanctithomae* | X | X | X |  |  |  | R |
| 9 | *Xiphocolaptes promeropirhynchus* |  | X |  |  |  |  | R |
| 5, 6, 8, 9, 10 | *Xiphorhynchus flavigaster* |  | X | M | M |  |  | R |
| 6 | *Xiphorhynchus erythropygius* | X | X | X | X |  |  | R |
| 4, 7 | *Lepidocolaptes leucogaster* | X | X | X |  |  |  | R |
| 6, 8 | *Lepidocolaptes souleyetii* |  | X | M | M |  |  | R |
| 6, 7, 8, 9 | *Lepidocolaptes affinis* |  | X | M | M |  |  | R |
| 10 | *Xenops minutus* | X |  |  |  |  |  | R |
| 8 | *Clibanornis rubiginosus* | X | X | X | X |  |  | R |
| 8, 9, 10 | *Synallaxis erythrothorax* |  | X | M | X |  |  | R |
|  | **Vireonidae** |  |  |  |  |  |  |  |
| 9, 10 | *Cyclarhis gujanensis* |  |  | X |  | X |  | R |
| 4, 7 | *Vireolanius melitophrys* | X | X |  | X | X |  | R |
| 6, 10 | *Pachysylvia decurtata* | X | X | X | X |  |  | R |
| 7, 8 | *Vireo hypochryseus* | X | X | X | X |  | X | R |
| 4, 7 | *Vireo brevipennis* | X | X |  |  |  |  | R |
| 4 | *Vireo nelsoni* | X | X |  |  |  |  | R |
| 5, 9, 10 | *Vireo griseus* |  |  | X |  |  | X | MI, R |
| 10 | *Vireo pallens* | X | X |  |  |  |  | R |
| 1, 2, 8 | *Vireo bellii* |  | X | X | X |  | X | MI, MV |
| 1, 3 | *Vireo vicinior* | X | X |  |  |  |  | MI, MV |
| 1, 2, 3, 4, 7, 8, 9 | *Vireo huttoni* |  |  | X | X | X | X | R |
| 10 | *Vireo flavifrons* |  |  |  |  |  |  | MI |
| 2, 4, 6, 7, 9 | *Vireo cassinii* | X |  | M | X |  |  | MI, MV, R |
| 5, 8 | *Vireo solitarius* |  |  | M | X |  | M | MI |
| 2, 4, 6, 7 | *Vireo plumbeus* |  | X |  |  |  |  | MI, R, MV |
| 2 | *Vireo philadelphicus* |  |  |  |  |  |  | MI |
| 2, 4, 5, 6, 7, 8, 9 | *Vireo gilvus* |  |  | M | M |  | M | MI, R |
| 6 | *Vireo leucophrys* |  | X | X | X |  |  | R |
| 8, 10 | *Vireo olivaceus* |  |  | X | X |  | X | T |
| 5, 9, 10 | *Vireo flavoviridis* |  |  | X |  |  | X | MV |
|  | **Laniidae** |  |  |  |  |  |  |  |
| 1, 2, 3, 4, 7 | *Lanius ludovicianus* |  | X | M |  | M |  | R, MI |
|  | **Corvidae** |  |  |  |  |  |  |  |
| 6, 8 | *Cyanolyca nanus* | X | X | M | M |  |  | R |
| 6, 9 | *Cyanolyca pumilo* | X | X | X |  |  |  | R |
| 6, 8, 9 | *Cyanolyca cucullata* | X | X | M | M |  |  | R |
| 2 | *Calocitta colliei* | X | X | X |  |  |  | R |
| 9 | *Calocitta formosa* |  |  |  |  |  |  | R |
| 5, 9, 10 | *Psilorhinus morio* |  |  | M | X | M |  | R |
| 5, 6, 8, 9, 10 | *Cyanocorax yncas* |  |  | M | M |  |  | R |
| 10 | *Cyanocorax yucatanicus* | X | X | X |  |  |  | R |
| 1, 2 | *Gymnorhinus cyanocephalus* |  | X |  |  |  |  | R |
| 2, 3, 4, 6, 7, 8, 9 | *Cyanocitta stelleri* |  |  | M | M | X |  | R |
| 1, 7 | *Aphelocoma californica* |  |  | M | X | X |  | R |
| 2, 3, 4, 6, 7, 8 | *Aphelocoma woodhouseii* |  | X | M | M | M |  | R |
| 4, 8 | *Aphelocoma ultramarina* | X | X | X | X |  |  | R |
| 2, 3, 10 | *Aphelocoma wollweberi* |  | X |  |  |  |  | R |
| 6, 8 | *Aphelocoma unicolor* | X | X | M | M |  |  | R |
| 2 | *Nucifraga columbiana* | X |  |  |  |  |  | R |
| 2 | *Corvus brachyrhynchos* |  |  |  |  |  |  | R |
| 2 | *Corvus cryptoleucus* |  |  |  |  |  |  | R, MI |
| 1, 2, 3, 4, 7, 8, 9 | *Corvus corax* |  |  | M | M | M | X | R |
|  | **Remizidae** |  |  |  |  |  |  |  |
| 1, 2, 3 | *Auriparus flaviceps* |  |  | M | X | M |  | R |
|  | **Paridae** |  |  |  |  |  |  |  |
| 1 | *Poecile gambeli* |  |  |  |  |  |  | T |
| 2, 4, 7 | *Poecile sclateri* | X | X | X |  |  |  | R |
| 2, 4, 7, 8 | *Baeolophus wollweberi* |  | X | M |  |  | X | R |
| 1 | *Baeolophus inornatus* |  | X |  |  |  |  | R |
|  | **Alaudidae** |  |  |  |  |  |  |  |
| 1, 2, 3, 4, 7 | *Eremophila alpestris* |  |  |  |  |  |  | R |
|  | **Hirundinidae** |  |  |  |  |  |  |  |
| 2 | *Riparia riparia* |  |  |  |  |  |  | T, MI, MV |
| 2, 3, 4, 9, 10 | *Tachycineta bicolor* |  |  |  |  |  |  | MI |
| 1, 2, 3, 4, 7, 8, 9 | *Tachycineta thalassina* |  |  | M | X | M | M | R, MI |
| 3, 8, 9, 10 | *Tachycineta albilinea* |  |  | M | X | M | X | R |
| 9 | *Atticora pileata* | X | X | X |  |  |  | R |
| 1, 2, 4, 6, 7, 8, 9, 10 | *Stelgidopteryx serripennis* |  |  | M | X | M | M | R, MI |
| 1, 2, 3, 4, 5, 10 | *Progne subis* |  |  | X |  | X |  | T, MV |
| 9, 10 | *Progne chalybea* |  |  |  |  |  |  | R, MV |
| 1, 2, 3, 4, 7, 8, 9, 10 | *Hirundo rustica* |  |  | M |  | M | M | MV, MI, R, T |
| 1, 2, 3, 4, 6, 7, 9 | *Petrochelidon pyrrhonota* |  |  | M | X | M | X | MV, T |
| 9 | *Petrochelidon fulva* |  |  |  |  |  |  | R, MV |
|  | **Aegithalidae** |  |  |  |  |  |  |  |
| 1, 2, 4, 6, 7, 8, 9 | *Psaltriparus minimus* |  |  | M | X | X | X | R |
|  | **Sylviidae** |  |  |  |  |  |  |  |
| 1 | *Chamaea fasciata* |  | X |  |  |  |  | R |
|  | **Zosteropidae** |  |  |  |  |  |  |  |
|  | **Regulidae** |  |  |  |  |  |  |  |
| 2, 3, 4,7, 8 | *Corthylio calendula* |  |  | X | X |  | X | MI |
| 4, 7 | *Regulus satrapa* |  |  |  |  |  |  | R, MI |
|  | **Bombycillidae** |  |  |  |  |  |  |  |
| 2, 4, 7 | *Bombycilla cedrorum* |  |  |  |  |  |  | MI |
|  | **Ptiliogonatidae** |  |  |  |  |  |  |  |
| 4, 5, 6, 7, 8, 9 | *Ptiliogonys cinereus* | X | X | M | M | X | X | R |
| 1, 2, 3, 4 | *Phainopepla nitens* |  |  | M |  |  | X | MI, R |
|  | **Sittidae** |  |  |  |  |  |  |  |
| 2 | *Sitta canadensis* |  |  |  |  |  |  | MI |
| 1, 4, 7 | *Sitta carolinensis* |  |  |  |  |  |  | R |
| 1, 4, 7 | *Sitta pygmaea* |  |  |  |  |  |  | R |
|  | **Certhiidae** |  |  |  |  |  |  |  |
| 2, 4, 7, 9 | *Certhia americana* |  |  |  |  |  |  | R, MI |
|  | **Polioptilidae** |  |  |  |  |  |  |  |
| 9, 10 | *Ramphocaenus melanurus* |  |  |  |  |  |  | R |
| 10 | *Polioptila bilineata* | X |  | X |  |  |  | R |
| 1, 3, 4, 5, 6, 7, 8, 9, 10 | *Polioptila caerulea* |  |  | M | M |  | X | MI, R |
| 1, 2, 3 | *Polioptila melanura* |  |  | X |  | X |  | R |
| 1, 3 | *Polioptila californica* |  | X |  |  |  |  | R |
| 3 | *Polioptila nigriceps* | X | X | X | X |  |  | R |
| 8, 9 | *Polioptila albiloris* |  | X | M | X |  | X | R |
|  | **Troglodytidae** |  |  |  |  |  |  |  |
| 1, 2, 3, 4, 7 | *Salpinctes obsoletus* |  |  | M |  | M |  | R |
| 1, 2, 3, 4, 5, 7, 8, 9 | *Catherpes mexicanus* |  |  | M | M | M | X | R |
| 5 | *Hylorchilus sumichrasti* | X | X | X |  | X |  | R |
| 5, 6, 8, 9, 10 | *Pheugopedius maculipectus* |  | X | M | M | X | M | R |
| 4, 8 | *Pheugopedius felix* | X | X | X | X | X | X | R |
| 9 | *Cantorchilus modestus* |  |  |  |  |  |  | R |
| 5, 6, 8, 10 | *Henicorhina leucosticta* |  | X | M | M | X | M | R |
| 4, 5, 6, 7, 8, 9 | *Henicorhina leucophrys* |  | X | M | M | X | M | R |
| 10 | *Uropsila leucogastra* | X | X |  |  |  |  | R |
| 4 | *Thryophilus sinaloa* | X | X |  |  |  |  | R |
| 9 | *Thryophilus pleurostictus* |  | X |  |  |  |  | R |
| 5, 6, 9 | *Campylorhynchus zonatus* |  |  | M | M |  |  | R |
| 4, 8 | *Campylorhynchus megalopterus* | X | X | M | M | X | X | R |
| 4 | *Campylorhynchus gularis* | X | X |  |  |  |  | R |
| 7, 8 | *Campylorhynchus jocosus* | X | X | M | X | X | X | R |
| 1, 2, 3 | *Campylorhynchus brunneicapillus* |  | X | M |  |  |  | R |
| 1, 2, 3, 4, 6, 7, 8 | *Thryomanes bewickii* |  |  | M | M | M | X | R |
| 10 | *Thryothorus ludovicianus* |  |  |  |  |  |  | R |
| 1, 2, 3, 4, 5, 6, 7, 8, 9, 10 | *Troglodytes aedon* |  |  | M | M | M | M | R, MI, T |
| 9, 10 | *Troglodytes rufociliatus* |  | X |  |  |  |  | R |
| 2 | *Troglodytes pacificus* |  |  |  |  |  |  | A |
| 4 | *Cistothorus platensis* |  |  |  |  |  |  | R, MI |
| 1, 2, 3, 4 | *Cistothorus palustris* |  |  |  |  |  |  | MI, R |
|  | **Mimidae** |  |  |  |  |  |  |  |
| 4, 5, 6, 7, 8 | *Melanotis caerulescens* | X | X | M | M | M | X | R |
| 9 | *Melanotis hypoleucus* |  | X | X |  |  |  | R |
| 10 | *Melanoptila glabrirostris* | X | X | X |  |  |  | R |
| 5, 9, 10 | *Dumetella carolinensis* |  |  | M | M |  |  | MI |
| 2, 3, 4, 7 | *Toxostoma curvirostre* |  |  | M | X | X |  | R |
| 4, 7 | *Toxostoma ocellatum* | X | X | M | X |  |  | R |
| 2, 3 | *Toxostoma bendirei* | X | X | X |  |  |  | R |
| 1 | *Toxostoma cinereum* | X | X | X |  |  |  | R |
| 1 | *Toxostoma redivivum* |  | X | X |  |  |  | R |
| 1, 2, 3 | *Toxostoma lecontei* |  | X | X |  |  |  | R |
| 1, 2, 3 | *Toxostoma crissale* |  | X | X |  |  |  | R |
| 2, 3 | *Oreoscoptes montanus* |  |  |  |  |  |  | MI |
| 9, 10 | *Mimus gilvus* |  |  | M | X |  | X | R |
| 1, 2, 3, 4, 7, 8 | *Mimus polyglottos* |  |  | M | M | M | X | R, MI |
|  | **Sturnidae** |  |  |  |  |  |  |  |
| 1, 2, 7 | *Sturnus vulgaris* |  |  |  |  |  |  | R |
|  | **Cinclidae** |  |  |  |  |  |  |  |
| 4, 6, 7, 8, 9 | *Cinclus mexicanus* | X | X | M | M |  | X | R |
|  | **Turdidae** |  |  |  |  |  |  |  |
| 4, 7, 8, 9 | *Sialia sialis* |  |  | M | M |  | X | MI, R |
| 1, 2, 4 | *Sialia mexicana* |  |  | X | X |  |  | R, MI |
| 2 | *Sialia currucoides* |  |  |  |  |  |  | MI |
| 2 | *Myadestes townsendi* | X |  |  |  |  |  | MI, R |
| 4, 5, 6, 7, 8, 9 | *Myadestes occidentalis* | X | X | M | M | X | X | R |
| 5, 6, 8 | *Myadestes unicolor* | X | X | M | M |  | X | R |
| 4, 5, 6, 7, 8, 9 | *Catharus aurantiirostris* |  | X | M | M | X | X | R, MI |
| 4, 6, 7, 8 | *Catharus occidentalis* | X | X | M | M | X | X | R |
| 4, 6, 7, 9 | *Catharus frantzii* | X | X | M | M | X |  | R |
| 5, 6, 8 | *Catharus mexicanus* | X | X | M | M |  | X | R |
| 6 | *Catharus fuscescens* |  | X | X | X |  |  | T |
| 2, 3, 7, 8, 9, 10 | *Catharus ustulatus* |  |  | X | X |  | X | T, MI |
| 1, 2, 3, 4, 6, 7, 8 | *Catharus guttatus* |  |  | M | M | X | X | MI |
| 5, 6, 9, 10 | *Hylocichla mustelina* |  | X | M | M |  |  | MI |
| 8, 9 | *Turdus infuscatus* | X | X | M | M |  | X | R |
| 9 | *Turdus plebejus* | X | X | X |  |  |  | R |
| 5, 6, 8, 9, 10 | *Turdus grayi* |  |  | M | M | X | X | R |
| 4, 5, 6, 7, 8 | *Turdus assimilis* |  | X | M | M |  | X | R |
| 4 | *Turdus rufopalliatus* | X |  |  |  |  |  | R |
| 9 | *Turdus rufitorques* | X | X | X |  |  |  | R |
| 2, 4, 7, 9 | *Turdus migratorius* |  |  | M | M | M | X | R, MI |
| 2 | *Ixoreus naevius* |  | X |  |  |  |  | MI |
| 4, 6, 7 | *Ridgwayia pinicola* | X | X |  |  |  |  | R |
|  | **Peucedramidae** |  |  |  |  |  |  |  |
| 2, 4, 7, 9 | *Peucedramus taeniatus* |  | X | M |  |  |  | R |
|  | **Passeridae** |  |  |  |  |  |  |  |
| 1, 2, 3, 4, 7, 8, 9 | *Passer domesticus* |  |  | M | X | X |  | R |
|  | **Motacillidae** |  |  |  |  |  |  |  |
| 2, 3, 4, 7 | *Anthus rubescens* |  |  |  |  |  |  | MI |
| 4 | *Anthus spragueii* |  | X |  |  |  |  | MI |
|  | **Fringillidae** |  |  |  |  |  |  |  |
| 4, 5, 6, 7 | *Chlorophonia elegantissima* |  | X | M | M |  | M | R |
| 6, 8 | *Chlorophonia occipitalis* |  | X | M | M |  | M | R |
| 5, 6, 8, 10 | *Euphonia affinis* |  |  | M | M |  | M | R |
| 5, 6, 9, 10 | *Euphonia hirundinacea* |  | X | M | M |  | M | R |
| 6 | *Euphonia gouldi* | X | X | X | X |  | X | R |
| 4, 6, 7, 8 | *Coccothraustes abeillei* | X | X | M | M |  | M | R |
| 2, 4, 7, 8 | *Coccothraustes vespertinus* |  | X | X | X |  | X | R |
| 1, 2, 3, 4, 7, 8 | *Haemorhous mexicanus* |  |  | M | M | X | X | R |
| 2 | *Haemorhous purpureus* |  |  |  |  |  |  | MI |
| 2 | *Haemorhous cassinii* |  | X |  |  |  |  | MI, R |
| 1, 4, 7, 9 | *Loxia curvirostra* |  |  |  |  |  |  | R |
| 1, 2, 4, 7, 9 | *Spinus spinus* |  |  | X |  |  | X | R, MI |
| 9 | *Spinus atriceps* | X | X | X |  |  | X | R |
| 4, 6, 7, 8, 9 | *Spinus notatus* |  | X | M | M |  | M | R |
| 1, 2, 3, 4, 5, 6, 7, 8, 9, 10 | *Spinus psaltria* |  |  | M | M |  | M | R |
| 1, 2 | *Spinus lawrencei* |  | X |  |  |  |  | MI, R |
| 2 | *Spinus tristis* |  |  |  |  |  |  | MI |
|  | **Calcariidae** |  |  |  |  |  |  |  |
| 2 | *Calcarius ornatus* |  | X |  |  |  |  | MI |
|  | **Passerellidae** |  |  |  |  |  |  |  |
| 5, 6, 7, 8, 9 | *Chlorospingus flavopectus* |  |  | M | M |  | M | R |
| 3 | *Peucaea carpalis* | X | X |  |  |  |  | R |
| 7, 8 | *Peucaea mystacalis* | X | X | X | X |  | X | R |
| 3, 4, 8 | *Peucaea botterii* |  | X | X | X |  | X | R |
| 3 | *Peucaea cassinii* |  |  |  |  |  |  | R, MI |
| 3, 4, 7 | *Ammodramus savannarum* |  | X |  |  |  |  | MI, R |
| 6, 10 | *Arremonops rufivirgatus* | X | X | M | M |  |  | R |
| 10 | *Arremonops chloronotus* |  | X | X | X |  |  | R |
| 3 | *Amphispizopsis quinquestriata* | X | X |  |  |  |  | R |
| 1, 2, 3 | *Amphispiza bilineata* |  |  |  |  |  |  | R |
| 1, 2, 3, 4 | *Chondestes grammacus* |  |  |  |  |  |  | MI, R |
| 2, 3 | *Calamospiza melanocorys* |  | X | X | X |  |  | MI |
| 1, 2, 3, 4, 7, 8 | *Spizella passerina* |  |  | M | X |  | X | R, MI |
| 4, 7 | *Spizella pallida* | X |  | X |  |  |  | MI |
| 1, 2 | *Spizella atrogularis* |  | X |  |  |  |  | R, MI |
| 2, 3 | *Spizella breweri* |  |  | X |  |  |  | MI |
| 4 | *Arremon virenticeps* | X | X |  |  |  |  | R |
| 5, 6, 7, 8 | *Arremon brunneinucha* |  | X | M | M |  | M | R |
| 1, 2 | *Junco hyemalis* |  |  |  |  |  |  | MI, R |
| 4, 6, 7, 9 | *Junco phaeonotus* | X |  | M | X |  |  | R |
| 9 | *Zonotrichia capensis* |  |  |  |  |  |  | R |
| 2, 3 | *Zonotrichia leucophrys* |  |  | M | X | X |  | MI |
| 2 | *Zonotrichia querula* |  | X |  |  |  |  | A |
| 3 | *Zonotrichia albicollis* |  |  |  |  |  |  | MI |
| 2 | *Artemisiospiza nevadensis* |  |  |  |  |  |  | MI |
| 1, 2, 3 | *Artemisiospiza belli* |  | X |  |  |  |  | R |
| 4 | *Oriturus superciliosus* | X | X |  |  |  |  | R |
| 2, 4, 7 | *Pooecetes gramineus* |  |  |  |  |  |  | MI |
| 1, 2, 3, 4, 8 | *Passerculus sandwichensis* |  |  | M | M |  | X | MI, R |
| 4 | *Xenospiza baileyi* | X | X |  |  |  |  | R |
| 1, 2, 3, 4 | *Melospiza melodia* |  |  |  |  |  |  | R, MI |
| 2, 3, 4, 5, 6, 7, 8, 9 | *Melospiza lincolnii* |  |  | M | M |  | M | MI |
| 2, 4 | *Melospiza georgiana* |  |  |  |  |  |  | MI |
| 4, 8 | *Melozone kieneri* | X | X | X | X |  | X | R |
| 2, 3, 4 | *Melozone fusca* |  |  |  |  |  |  | R |
| 7, 8 | *Melozone albicollis* | X | X | M | M | X | X | R |
| 1, 2 | *Melozone aberti* |  | X | X | X | X |  | R |
| 1 | *Melozone crissalis* |  |  |  |  |  |  | R |
| 9 | *Melozone biarcuata* | X |  | X |  |  |  | R |
| 4, 5, 9 | *Aimophila rufescens* |  |  | X | X |  | X | R |
| 1, 2, 4, 6, 7, 8 | *Aimophila ruficeps* |  |  | M | M |  | X | R |
| 8 | *Aimophila notosticta* | X | X | X | X |  | X | R |
| 1, 3, 4 | *Pipilo chlorurus* |  | X | X |  |  |  | MI, R |
| 1, 2, 3, 4, 7, 8, 9 | *Pipilo maculatus* |  |  | M |  | M | X | R, MI |
| 7 | *Pipilo ocai* | X | X | X |  |  |  | R |
| 4, 7, 8 | *Atlapetes pileatus* | X | X | M | X |  | X | R |
| 5, 9 | *Atlapetes albinucha* |  | X | M | M | X | X | R |
|  | **Icteriidae** |  |  |  |  |  |  |  |
| 1, 2, 3, 4, 8, 9, 10 | *Icteria virens* |  |  | M | M |  | X | MI, MV |
|  | **Icteridae** |  |  |  |  |  |  |  |
| 1, 2, 4 | *Xanthocephalus xanthocephalus* |  |  |  |  |  |  | MI |
| 2, 4, 9, | *Sturnella magna* |  |  |  |  |  |  | R |
| 1,2,3, | *Sturnella neglecta* |  |  | X |  |  | X | R, MI |
| 5, 6, 9, 10 | *Amblycercus holosericeus* |  | X | M | M | X | M | R |
| 5, 9, 10 | *Psarocolius wagleri* | X | X | M | X |  |  | R |
| 5, 9, 10 | *Psarocolius montezuma* | X | X | M | M |  |  | R |
| 2, 4, 5, 7, 8, 9 | *Icterus wagleri* |  | X | M | X | M |  | R |
| 6, 10 | *Icterus prosthemelas* |  | X | M | X |  | X | R |
| 2, 4, 7, 9, 10 | *Icterus spurius* |  |  | M |  | M |  | MI, MV |
| 1, 2, 3, 4, 10 | *Icterus cucullatus* | X |  | M | X | X |  | MI, MV, R |
| 9, 10 | *Icterus chrysater* |  | X | M |  | X |  | R |
| 9, 10 | *Icterus mesomelas* |  | X | M |  | X |  | R |
| 3, 4, 7, 9 | *Icterus pustulatus* |  |  | X |  | X |  | R |
| 1, 2, 4, 7 | *Icterus bullockii* | X |  | M | X | M |  | MI, MV |
| 10 | *Icterus auratus* | X | X | X |  |  |  | R |
| 5, 8, 10 | *Icterus gularis* |  |  | M | M |  |  | R |
| 5, 6, 7, 8 | *Icterus graduacauda* | X | X | M | M | X | X | R |
| 3, 5, 6, 8, 9, 10 | *Icterus galbula* |  |  | M | M | X | X | MI |
| 1, 2, 3, 4 | *Icterus parisorum* | X | X | X | X | X |  | R, MV, MI |
| 1, 2, 4, 9, 10 | *Agelaius phoeniceus* |  |  | X |  | X |  | R, MI |
| 1 | *Agelaius tricolor* |  | X |  |  |  |  | R |
| 2, 3, 4, 5, 6, 7, 8, 9, 10 | *Molothrus aeneus* |  |  | M | M | X |  | R, MV |
| 1, 2, 3, 4, 6 | *Molothrus ater* |  |  | X | X |  |  | R, MI |
| 10 | *Molothrus oryzivorus* |  |  |  |  |  |  | R |
| 5, 6, 9, 10 | *Dives dives* |  |  | X | X |  | X | R |
| 1, 2, 3, 4 | *Euphagus cyanocephalus* |  |  |  |  |  |  | MI, R |
| 1, 2, 3, 4, 5, 7, 9, 10 | *Quiscalus mexicanus* |  |  | M | X |  |  | R |
|  | **Parulidae** |  |  |  |  |  |  |  |
| 2, 3, 4, 5, 6, 8, 9, 10 | *Seiurus aurocapilla* |  |  | M | M |  | X | MI |
| 5, 10 | *Helmitheros vermivorum* |  | X | X | X |  |  | MI |
| 5, 6, 7, 9 | *Parkesia motacilla* |  | X | M | M |  |  | MI |
| 3, 4, 9, 10 | *Parkesia noveboracensis* |  |  |  |  |  |  | MI |
| 5, 10 | *Vermivora cyanoptera* |  | X | X | X |  |  | MI |
| 2, 3, 4, 5, 6, 7, 8, 9, 10 | *Mniotilta varia* |  |  | M | M |  | M | MI |
| 2, 10 | *Protonotaria citrea* |  | X |  |  |  |  | MI |
| 10 | *Limnothlypis swainsonii* |  | X |  |  |  |  | MI |
| 4, 7, 9 | *Oreothlypis superciliosa* |  | X | M |  |  |  | R |
| 2, 5, 8, 9, 10 | *Leiothlypis peregrina* |  |  | M | X |  | M | MI |
| 1, 2, 3, 4, 5, 7, 8, 9 | *Leiothlypis celata* |  |  | M | X |  | M | MI, R |
| 4 | *Leiothlypis crissalis* | X | X |  |  |  |  | MI, MV |
| 1, 2 | *Leiothlypis luciae* | X |  |  |  |  |  | MI, MV |
| 2, 3, 4, 5, 6, 7, 8, 9 | *Leiothlypis ruficapilla* |  |  | M | M |  | M | MI |
| 2, 4, 8 | *Leiothlypis virginiae* | X | X | X | X |  | X | MI |
| 4, 9 | *Geothlypis poliocephala* |  |  |  |  |  |  | R |
| 2, 3, 4, 5, 6, 7, 8, 9 | *Geothlypis tolmiei* | X | X | M | M |  | M | MI |
| 9, 10 | *Geothlypis philadelphia* |  | X |  |  |  |  | T, MI |
| 8 | *Geothlypis formosa* |  | X | X | X |  | X | MI |
| 4 | *Geothlypis speciosa* | X | X |  |  |  |  | R |
| 1, 2, 4, 5, 8, 9, 10 | *Geothlypis trichas* |  |  | M | M |  | M | MI, R |
| 5, 8, 10 | *Setophaga citrina* |  |  | M | X |  | M | MI |
| 2, 5, 10 | *Setophaga ruticilla* |  |  | X |  |  | X | MI |
| 2, 8, 10 | *Setophaga americana* |  |  | X | X |  | X | MI |
| 6, 9 | *Setophaga pitiayumi* |  |  | X | X |  |  | R, MI |
| 5, 6, 8, 9, 10 | *Setophaga magnolia* |  |  | M | M |  | M | MI |
| 9 | *Setophaga fusca* |  |  |  |  |  |  | T |
| 1, 2, 3, 4, 9, 10 | *Setophaga petechia* |  |  |  |  |  |  | MI, MV, T, R |
| 2, 9 | *Setophaga pensylvanica* |  | X |  |  |  |  | T, MI |
| 9 | *Setophaga caerulescens* |  |  |  |  |  |  | MI |
| 2, 3, 4, 5, 7, 8, 9, 10 | *Setophaga coronata* |  |  | M | X | X | M | MI, R |
| 5, 7, 9, 10 | *Setophaga dominica* |  |  | X |  |  | X | MI |
| 2, 4, 9 | *Setophaga graciae* |  | X |  |  |  |  | R, MV |
| 1, 2, 3, 4, 7, 8 | *Setophaga nigrescens* | X | X | X | X |  | X | MI |
| 2, 4, 5, 6, 7, 8, 9 | *Setophaga townsendi* |  | X | M | M | X | M | MI |
| 2, 4, 6, 7, 9 | *Setophaga occidentalis* |  | X | M | X |  |  | MI |
| 8, 9 | *Setophaga chrysoparia* | X | X | M | X |  | X | T, MI |
| 5, 6, 7, 8, 9, 10 | *Setophaga virens* |  |  | M | M |  | M | MI |
| 5, 6, 8 | *Basileuterus lachrymosus* |  | X | M | M | X | M | R |
| 4, 5, 6, 7, 8, 9 | *Basileuterus rufifrons* | X | X | M | M | M | M | R |
| 4, 5, 6, 7, 8, 9, 10 | *Basileuterus belli* |  | X | M | M | X | M | R |
| 5, 6, 8 | *Basileuterus culicivorus* |  |  | M | M | X | X | R |
| 9 | *Cardellina canadensis* |  | X | X |  |  |  | T |
| 1, 2, 3, 4, 5, 6, 7, 8, 9 | *Cardellina pusilla* |  |  | M | M | M | M | MI |
| 4, 7, 8 | *Cardellina rubrifrons* | X | X | X | X | X | X | MI, MV |
| 4, 6, 7, 8 | *Cardellina rubra* | X | X | M | M | M | X | R |
| 9 | *Cardellina versicolor* | X | X | X |  |  |  | R |
| 4, 6, 7, 8, 9 | *Myioborus pictus* |  | X | M | M | X | X | R |
| 4, 5, 6, 7, 8, 9 | *Myioborus miniatus* |  |  | M | M | X | M | R |
|  | **Cardinalidae** |  |  |  |  |  |  |  |
| 4, 6, 7, 8, 9 | *Piranga flava* |  |  | M | M | M | X | R |
| 1, 2, 4, 5, 7, 8, 9, 10 | *Piranga rubra* |  |  | M | M | M | X | MI, MV |
| 10 | *Piranga olivacea* |  | X |  |  |  |  | T |
| 1, 2, 3, 4, 7, 8 | *Piranga ludoviciana* |  |  | M | X | M | X | MI |
| 4, 5, 8 | *Piranga bidentata* |  | X | M | M | X | X | R |
| 5, 6 | *Piranga leucoptera* |  | X | M | M | X | X | R |
| 7, 8 | *Piranga erythrocephala* | X | X |  |  |  |  | R |
| 6, 7, 8, 10 | *Habia rubica* |  |  | M | M | M | X | R |
| 5, 6, 8, 9, 10 | *Habia fuscicauda* |  | X | M | M | X | X | R |
| 6, 8 | *Caryothraustes poliogaster* |  | X | M | M | X | X | R |
| 2, 3, 4, 10 | *Cardinalis cardinalis* |  |  | M | M | M | X | R |
| 2, 3 | *Cardinalis sinuatus* |  | X | X |  |  |  | R |
| 4, 6, 8, 9 | *Pheucticus chrysopeplus* | X | X | M | M | X | X | R |
| 2, 3, 5, 8, 9, 10 | *Pheucticus ludovicianus* |  |  | M | M | X | X | MI, T |
| 1, 2, 3, 4, 5, 7 | *Pheucticus melanocephalus* | X |  | M | M | M | X | R, MI, MV |
| 10 | *Granatellus sallaei* |  | X | X |  |  |  | R |
| 6 | *Cyanoloxia cyanoides* |  |  | X | X |  |  | R |
| 5, 6, 8, 10 | *Cyanocompsa parellina* |  | X | M | M | X | X | R |
| 1, 2, 4, 7, 8, 10 | *Passerina caerulea* |  |  | M | M | M | X | MI, R, MV |
| 1, 2, 3, 4, 8 | *Passerina amoena* | X |  | M | X | X | X | MI, MV |
| 2, 7, 8, 10 | *Passerina cyanea* |  |  | M | M | X | X | MI |
| 3, 4 | *Passerina versicolor* |  | X |  |  |  |  | R, MV |
| 8, 10 | *Passerina ciris* | X | X | M | M | X | X | MI, MV |
|  | **Thraupidae** |  |  |  |  |  |  |  |
| 5, 6, 8, 10 | *Thraupis episcopus* |  |  | M | M | X | M | R |
| 5, 6, 8, 9, 10 | *Thraupis abbas* |  |  | M | M | X | M | R |
| 6 | *Stilpnia larvata* |  | X | X | X |  |  | R |
| 4, 5, 6, 7, 8, 9 | *Diglossa baritula* |  | X | M | M |  | X | R |
| 6, 8 | *Chlorophanes spiza* |  | X | M | M |  | X | R |
| 6, 9, 10 | *Volatinia jacarina* |  |  | M | X |  |  | R |
| 10 | *Eucometis penicillata* | X | X |  |  |  |  | R |
| 5, 6, 10 | *Lanio aurantius* | X | X | M | M |  |  | R |
| 5, 6, 9 | *Ramphocelus sanguinolentus* |  | X | M | M |  |  | R |
| 5, 6, 8, 10 | *Cyanerpes cyaneus* |  |  | M | M |  | X | R |
| 5, 6, 8 | *Coereba flaveola* |  |  | M | M |  | X | R |
| 5, 6, 8, 9, 10 | *Tiaris olivaceus* |  |  | M | M |  | X | R |
| 6 | *Sporophila corvina* |  |  | X | X |  |  | R |
| 4, 7, 8, 9 | *Sporophila torqueola* | X |  | X |  |  | X | R |
| 5, 6, 9, 10 | *Sporophila morelleti* |  |  | M | M |  | X | R |
| 5, 6, 9, 10 | *Saltator atriceps* |  |  | X | X |  | X | R |
| 5, 6, 8 | *Saltator maximus* |  |  | M | M |  | M | R |
| 5, 6, 9, 10 | *Saltator coerulescens* |  |  | X | X |  | X | R |
